# Whole genome mutagenicity evaluation using Hawk-Seq™ demonstrates high inter-laboratory reproducibility and concordance with the transgenic rodent gene mutation assay

**DOI:** 10.1186/s41021-025-00336-w

**Published:** 2025-07-29

**Authors:** Shoji Matsumura, Sayaka Hosoi, Takako Hirose, Yuki Otsubo, Kazutoshi Saito, Masaaki Miyazawa, Akihiro Kawade, Atsushi Hakura, Dai Kakiuchi, Shoji Asakura, Naoki Koyama, Yuki Okada, Satsuki Chikura, Takafumi Kimoto, Kenichi Masumura, Takayoshi Suzuki, Kei-ichi Sugiyama

**Affiliations:** 1 R&D -Safety Science Research, Kao Corporation, 3-25-14 Tonomachi, Kawasaki-ku, Kawasaki-shi, Kanagawa 210-0821 Japan; 2 R&D -Safety Science Research, Kao Corporation, 2606 Akabane, Ichikai-Machi, Haga-Gun, Tochigi, 321-3497 Japan; 3 Drug Safety & Animal Care Technology Unit, Tsukuba Division, Sunplanet Co., Ltd, 5-1-3 Tokodai, Tsukuba-shi, Ibaraki 300-2635 Japan; 4 Global Drug Safety, Eisai Co., Ltd, 5-1-3 Tokodai, Tsukuba-shi, Ibaraki 300-2635 Japan; 5 Translational Research Division, Safety and Bioscience Research Department, Chugai Pharmaceutical Co., Ltd, 216 Totsuka, Totsuka-ku, Yokohama-shi, Kanagawa 244- 8602 Japan; 6 Teijin Pharma Limited, 4-3-2 Asahigaoka, Hino, Tokyo, 191-8512 Japan; 7 Division of Risk Assessment, National Institute of Health Sciences, 3-25-26 Tonomachi, Kawasaki-ku, Kawasaki-shi, Kanagawa 210-9501 Japan; 8 Division of Genome Safety Science, National Institute of Health Sciences, 3-25-26 Tonomachi, Kawasaki-ku, Kawasaki-shi, Kanagawa 210-9501 Japan

**Keywords:** Error-corrected next-generation sequencing, Hawk-Seq™, Mutagenicity assay, Transgenic rodent model, Validation study, Reproducibility, Transferability

## Abstract

**Background:**

Error-corrected next-generation sequencing (ecNGS) enables the sensitive detection of chemically induced mutations. Matsumura et al. reported Hawk-Seq™, an ecNGS method, demonstrating its utility in clarifying mutagenicity both qualitatively and quantitatively. To further promote the adoption of ecNGS-based assays, it is important to evaluate their inter-laboratory transferability and reproducibility. Therefore, we evaluated the inter-laboratory reproducibility of Hawk-Seq™ and its concordance with the transgenic rodent mutation (TGR) assay.

**Results:**

The Hawk-Seq™ protocol was successfully transferred from the developer’s laboratory (lab A) to two additional laboratories (labs B, C). Whole genomic mutations were analyzed independently using the same genomic DNA samples from the livers of *gpt* delta mice exposed to benzo[*a*]pyrene (BP), *N*-ethyl-*N*-nitrosourea (ENU), and *N*-methyl-*N*-nitrosourea (MNU). In all laboratories, clear dose-dependent increases in base substitution (BS) frequencies were observed, specific to each mutagen (e.g. G:C to T:A for BP). Statistically significant increases in overall mutation frequencies (OMFs) were identified at the same doses across all laboratories, suggesting high reproducibility in mutagenicity assessment. The correlation coefficient (r^2^) of the six types of BS frequencies exceeded 0.97 among the three laboratories for BP- or ENU-exposed samples. Thus, Hawk-Seq™ provides qualitatively and quantitatively reproducible results across laboratories. The OMFs in the Hawk-Seq™ analysis positively correlated (r^2^ = 0.64) with *gpt* mutant frequencies (MFs). The fold induction of OMFs in the Hawk-Seq™ analysis of ENU- and MNU-exposed samples was at least 14.2 and 4.5, respectively, compared to 6.1 and 2.5 for *gpt* MFs. Meanwhile, the fold induction of OMFs in BP-exposed samples was ≤ 4.6, compared to 8.2 for *gpt* MFs. These observations suggest that Hawk-Seq™ demonstrates good concordance with the transgenic rodent (TGR) gene mutation assay, whereas the induction of mutation frequency by each mutagen might not directly correspond.

**Conclusions:**

Hawk-Seq™-based whole-genome mutagenicity evaluation demonstrated high inter-laboratory reproducibility and concordance with *gpt* assay results. Our results contribute to the growing evidence that ecNGS assays provide a suitable, or improved, alternative to the TGR assay.

**Supplementary Information:**

The online version contains supplementary material available at 10.1186/s41021-025-00336-w.

## Introduction

Error-corrected next-generation sequencing (ecNGS) technologies can dramatically reduce the error frequency in next-generation sequencing (NGS) by utilizing complementary strand information, thereby enabling the direct detection of mutagen-induced mutations in genomic DNA [1–4]. Since their introduction in the early 2010s, several ecNGS methodologies have been developed, and their utility in detecting rare genomic mutations has been demonstrated [5–14]. Recently, we reported an ecNGS methodology, Hawk-Seq™, and demonstrated its utility in obtaining mutation data that reflect the mechanisms of mutagens [10, 15]. These ecNGS methods can clarify induced genomic mutations with sufficient resolution to identify mutagen-specific features.

The utility of ecNGS methodologies for detecting mutations has been demonstrated in several studies [9–11, 13–17]. These studies proved that ecNGS can detect mutations in bacterial [9, 10, 15, 16], *in vivo* rodent [9, 14, 17], and *in vitro* mammalian cell culture models [11, 18, 19]. Most of these studies detected the induced mutations caused by agents such as benzo[*a*]pyrene (BP) and *N-*ethyl*-N-*nitrosourea (ENU) and demonstrated sufficient sensitivity to comprehend substance-specific mutational features. Each method differs from the others in terms of the theory and protocol, for detecting mutations, and they have distinct strengths and limitations for analyzing mutagen-induced mutations [3]. Therefore, the concurrent standardization of multiple methodologies would be highly beneficial to the field of chemical genotoxicity research.

To promote the adoption of new methods, the OECD recommends validation studies, including the evaluation of their inter-laboratory transferability and reproducibility, are important [20]. Although the inter-laboratory reproducibility of duplex sequencing has been evaluated in several experimental models [21–23], similar studies on other ecNGS methodologies have not been performed. To promote acceptance and elevate the universality and applicability of ecNGS methodologies, the transferability and inter-laboratory reproducibility of other methods should be evaluated.

Thus, we evaluated the inter-laboratory reproducibility of mutagenicity evaluation by Hawk-Seq™ using the same DNA samples analyzed at three facilities, as a collaborative study within the Japanese Environmental Mutagen and Genome Society (JEMS)–Mammalian Mutagenicity Study Group (MMS). Kao (lab A) transferred the Hawk-Seq™ experimental protocols to Teijin Pharma Limited (lab B) and Eisai Co., Ltd. (lab C). The DNA samples were from a previous experiment in which *gpt* delta mice were exposed to mutagens. Three laboratories independently prepared sequencing libraries for Hawk-Seq™ using the same DNA samples and analyzed the induced mutations. By evaluating the reproducibility of results in terms of assay efficiency, mutation frequency, and outcomes of statistical analysis, we confirmed that Hawk-Seq™, an ecNGS-based assay, can provide reproducible results between laboratories. Concordance with the transgenic rodent (TGR) gene mutation assay was confirmed by comparing mutation frequencies.

## Materials and methods

### Library preparation and sequencing

The 32 genomic DNA samples of the livers from male inbred C57BL/6JJmsSlc-Tg (*gpt* delta) mice (7–9 weeks old) prepared in our previous study were used [9]. The group compositions and *gpt* mutant frequencies obtained in the previous study are summarized in Table 1 [9]. Briefly, BP (CASRN. 50-32-8) was administered orally to mice once daily for 5 days at 0 (olive oil), 150, and 300 mg/kg. Meanwhile, ENU (CASRN. 759-73-9) was administered intraperitoneally once daily for 5 days at 0 (saline), 75, and 150 mg/kg. *N*-Methyl-*N*-nitrosourea (MNU; CAS RN. 684-93-5) was administered intraperitoneally once daily for 5 days at 0 (saline), 12.5, and 25 mg/kg. ENU and MNU shared the same vehicle control group. Genomic DNAs were extracted from the liver 7 days after the final treatment using the RecoverEase DNA Isolation Kit (Agilent Technologies, CA, USA) and stored in a freezer. These DNA samples were shipped to each laboratory, where libraries for Hawk-Seq™ analysis were prepared as previously described (Supplementary Materials and Methods) [9]. Briefly, 60 ng of genomic DNA was transferred to Covaris tubes and sheared into fragments with a peak size of 350 bp using a sonicator (Covaris, MA, USA). The resulting DNA fragments were used for sequencing library preparation using the TruSeq Nano DNA Low-Throughput Library Prep Kit (TruSeq; Illumina, San Diego, CA, USA). The sonicated DNA fragments were subjected to end repair, 3ʹ dA-tailing, and ligation to TruSeq-indexed adapters, according to the manufacturer’s instructions. The DNA concentration of each ligated sample was measured using either an Agilent 4200 TapeStation or Agilent 2100 Bioanalyzer (Agilent Technologies, CA, USA). To determine the optimal DNA amount subjected to PCR, a series of dilutions of ligated products was prepared with concentrations ranging from 0.8 to 25 amol/µL, and 25 µL of each sample was used for PCR enrichment (Supplementary Table 1). For the analysis of mutagen-exposed samples, the ligated products were diluted to 3.1 amol/µL (lab A) or 6.2 amol/µL (labs B and C) with suspension buffer, and 25 µL of these diluted products were subjected to PCR amplification to prepare sequencing libraries. The obtained libraries were sequenced with 2 × 151 bp using NovaSeq6000 (NovaSeq; Illumina, CA, USA) to yield at least 50 M paired-end reads.

**Table 1:** Group composition and *gpt* mutant frequencies of liver DNA samples used in this study [9]

Mutagens	Dose (mg/kg/day)	Dosing period (days)	Animal No.	*gpt* mutant frequency (×10^−6^, Mean $\:\pm\:$ SD)
BP (p.o.)	0 (Olive oil)	5	1002, 1003, 1004, 1005	2.43 $\:\pm\:$ 0.81
	150		1201, 1203, 1204, 1205	9.14 $\:\pm\:$ 2.35 ^S^
	300		1301, 1302, 1303, 1305	19.91 $\:\pm\:$ 9.79 ^S^
ENU (i.p.)	0 (Saline)	5	3001, 6001, 6002, 6003	3.85 $\:\pm\:$ 3.19
	75		3201, 3202, 6201, 6203	6.61 $\:\pm\:$ 2.73
	150		3302, 6301, 6302, 6303	23.48 $\:\pm\:$ 18.58 ^S^
MNU (i.p.)	12.5	5	5101, 5102, 8101, 8102	4.5 $\:\pm\:$ 1.21
	25		5202, 8201, 8202, 8203	9.6 $\:\pm\:$ 4.03 ^D^

*^S^: *p* < 0.05 by Steel’s test, *^D^: *p* < 0.05 by Dunnett’s test. ENU and MNU share the same vehicle control group

### Processing of sequencing data

Data analysis pipelines were implemented independently in each laboratory following the procedure described below. Adaptor sequences and low-quality bases were removed from the generated paired-end reads using Cutadapt-3.5 with the following options: -a AGATCGGAAGAGCAC, -A AGATCGGAAGAGCGT, -e 0.3, -O 3, -u −20, -U −20, -q 33, -m 50, -n 2, --max-ee = 0.5, and --max-n = 0 [23]. The edited paired-end reads were mapped to the GRCm38 mouse reference genome sequence using Bowtie2-2.4.1 [24]. SAM format processing was performed using SAMtools-1.10 [25]. Read pairs were limited to uniquely mapped reads by excluding those with a MAPQ score ≤ 41 or those containing the “XS:i” tag. To prepare double-stranded DNA consensus sequences (dsDCS), read pairs that shared the same genomic positions (start and end positions on the reference genome) were grouped into the same position groups (SP-Gs) and divided into two subgroups based on their R1 and R2 orientations. SP-Gs containing read pairs in both orientations were identified and used to generate dsDCS read pairs [9]. A dsDCS read pair was generated from an SP-G if at least one read pair from each strand was present. The resulting dsDCS read pairs were mapped to the reference genome sequence using Bowtie2-2.4.1. The obtained SAM files were processed using SAMtools, and mutations were detected. To calculate the efficiency of obtaining dsDCS read pairs, the number of dsDCS read pairs was divided by the number of read pairs sequenced for each sample.

### Calculation of mutation frequency and reproducibility evaluation

To calculate the mutation frequency, the number of base substitutions for each type was enumerated separately. The frequency for each substitution type per 10^6^ G:C or A:T base pairs was calculated by dividing each mutation count by the total dsDCS read base count mapped to G:C or A:T base pair. The overall mutation frequency (OMF) was calculated by dividing the total mutation count by the total dsDCS read base count. To reduce background mutation calls caused by single nucleotide polymorphisms (SNPs), genomic positions listed in the Ensembl variation database (version 102) were removed from the analysis [26]. In addition, suspected variant positions of *gpt* delta mice, which redundantly denoted mutations in two or more samples in our laboratory historical data (*n* = 20 of vehicle controls), were excluded from the analysis [9]. Supplementary Fig. 2 indicates that 12 or more historical data points (Supplementary Table 2) were sufficient to reduce the background error frequency to the order of 10^−7^ bp under our experimental conditions. Statistical analyses were performed based on the frequencies of each base substitution type and overall mutations. First, Bartlett’s test for homogeneity of variance was performed. If homogeneity was determined (not significant on Bartlett’s test), Dunnett’s multiple comparison test was performed. If no homogeneity (significant on Bartlett’s test) was present, Steel’s test (two-sided, significance level of 0.05) was performed.

## Results

### Technical transfer of library preparation protocol

During library preparation for Hawk-Seq™, the concentrations of adapter-ligated products and PCR-amplified final libraries were determined to optimize the efficiency of generating dsDCS reads per unit sequencing amount, increasing assay efficiency and reproducibility. We first evaluated the applicability of two instruments used in the collaborative laboratories (4200 TapeStation and 2100 Bioanalyzer) for determining DNA concentration. Our data indicate these two instruments are equally applicable for determining the concentrations of library products (Supplementary Fig. 1). Table 2 shows the instruments used for library preparation in each laboratory. All laboratories used the NovaSeq 6000 platform, because differences in sequencing platforms could affect the background error frequency under Hawk-Seq™ analysis [27]. During the transfer of the library preparation protocol, the amount of DNA subjected to PCR enrichment was optimized in each laboratory. Supplementary Table 1 shows the efficiency of dsDCS yields depending on the amount of input DNA used in each laboratory experiment. The highest efficiency was observed at 156 amol in the lab B and lab C (both indicated 9%). Meanwhile, lab A was indicated at 78 amol (7%) in a previous study [9]. Subsequent analyses of mutagen-exposed samples were performed under these conditions.

**Table 2:** Instruments used for library preparation in each laboratory

Step	Lab A	Lab B	Lab C
DNA concentration measurement	Qubit Flex Fluorometer	Qubit Flex Fluorometer	Qubit Flex Fluorometer
DNA fragmentation	Covaris ME220	Covaris M220	Covaris ME220
QC for sequencing library	4200 TapeStation	2100 BioAnalyzer	2100 BioAnalyzer
Sequencing	NovaSeq 6000	NovaSeq 6000	NovaSeq 6000

### Mutation frequencies and spectra induced by mutagens

**Fig. 1:**
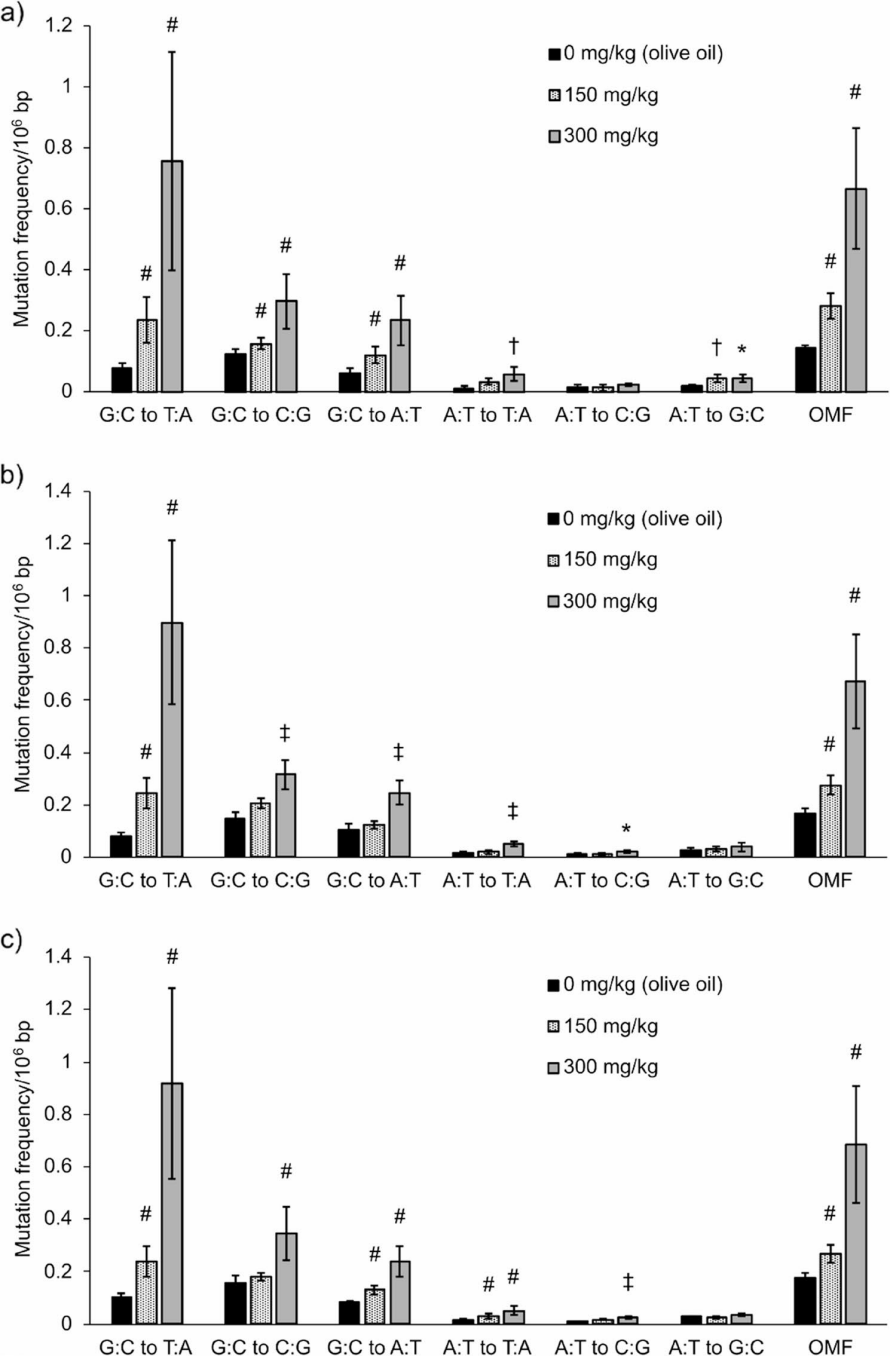
The mean frequencies of six types of base substitutions and OMFs in BP-exposed samples (*n* = 4) in (**a**) lab A, (**b**) lab B, and (**c**) lab C. Error bars indicate standard deviation. A statistically significant increase in OMFs was identified at 150 and 300 mg/kg in all laboratories. Asterisks, daggers, and hashes indicate p-values in Dunnett’s or Steel’s multiple comparison tests (* *p* < 0.05, † *p* < 0.01, and ‡ *p* < 0.001, respectively, by Dunnett’s test. #: *p* < 0.05 by Steel’s test)

**Fig. 2:**
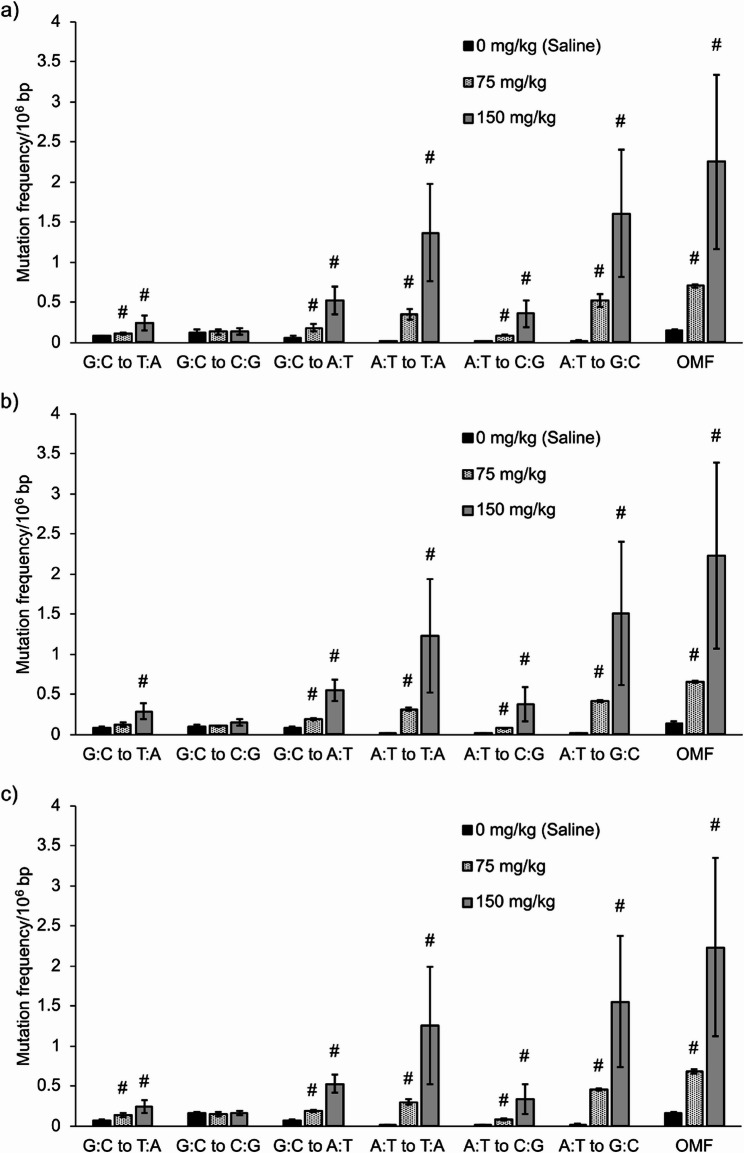
The mean frequencies of six types of base substitutions and OMFs in ENU-exposed samples (*n* = 4) in (**a**) lab A, (**b**) lab B, and (**c**) lab C. Error bars indicate standard deviation. A statistically significant increase in OMF was identified at 75 and 150 mg/kg in all laboratories. Asterisks, daggers, and hashes indicate p-values in Dunnett’s or Steel’s multiple comparison tests (* *p* < 0.05, † *p* < 0.01, and ‡ *p* < 0.001, respectively, by Dunnett’s test. #: *p* < 0.05 by Steel’s test)

**Fig. 3:**
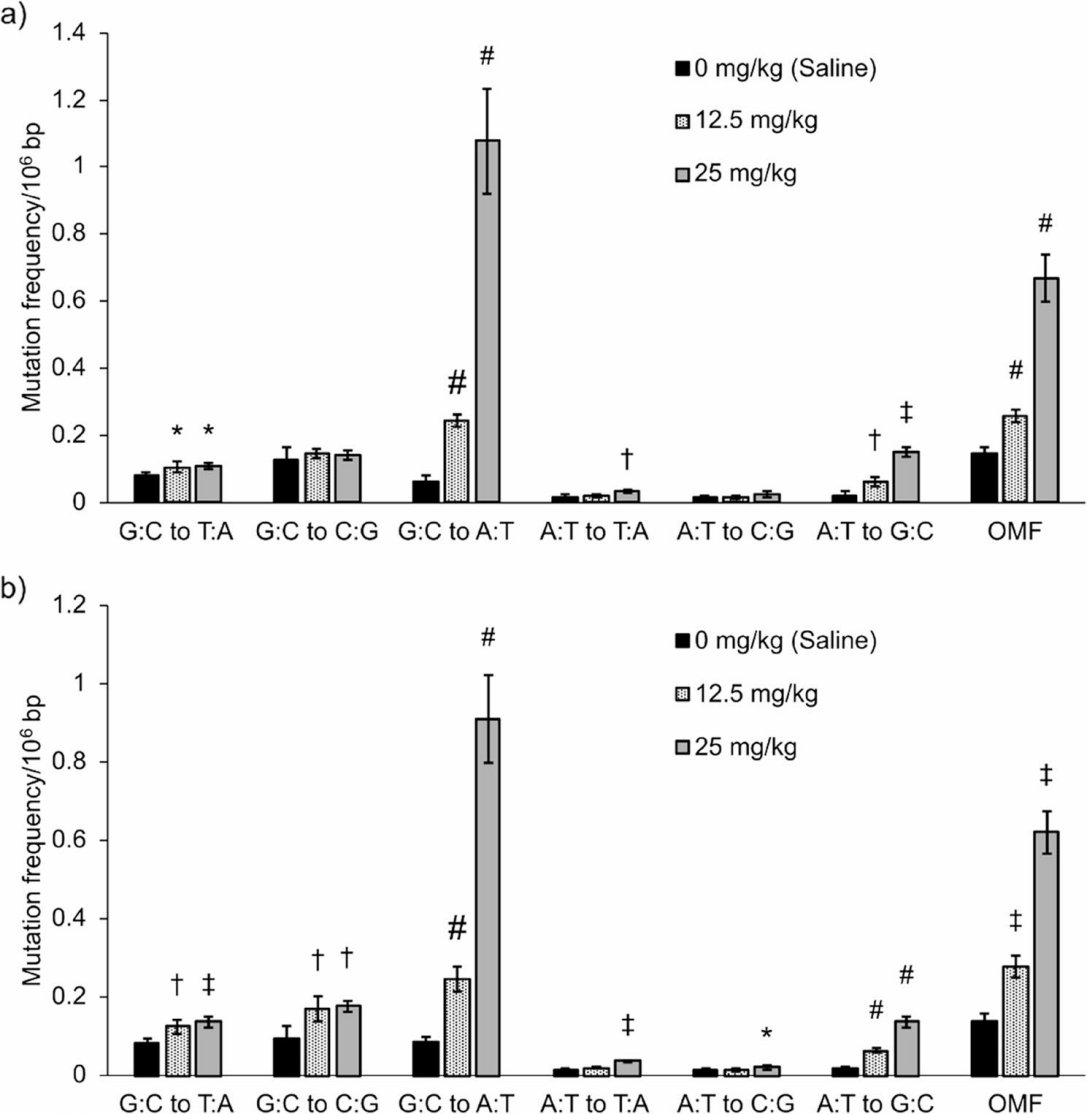
Mean frequencies of six types of base substitutions and OMFs in MNU-exposed samples (*n* = 4) in (**a**) lab A and (**b**) lab B. Error bars indicate standard deviation. A statistically significant increase in OMF was identified at 12.5 and 25 mg/kg in both laboratories. Asterisks, daggers, and hashes indicate p-values in Dunnett’s or Steel’s multiple comparison tests (* *p* < 0.05, † *p* < 0.01, and ‡ *p* < 0.001, respectively, by Dunnett’s test. #: *p* < 0.05 by Steel’s test)

First, the mean ± SD of total analyzable dsDCS bases across all samples (32 for labs A and B, 24 for lab C) were 9.0 ± 1.1 × 10^8^ bp, 8.7 ± 2.9 × 10^8^ bp, and 18.1 ± 3.6 × 10^8^ bp in labs A, B, and C, respectively. The mean frequencies of six types of base substitutions and OMFs in BP-exposed samples across all laboratories are shown in Fig. 1. The mean OMFs from lab A, B, and C were similar: 0.144 × 10^−6^, 0.169 × 10^−6^, and 0.174 × 10^−6^ bp for olive oil, 0.283 × 10^−6^, 0.276 × 10^−6^, and 0.269 × 10^−6^ bp for 150 mg/kg, 0.666 × 10^−6^, 0.672 × 10^−6^, and 0.685 × 10^−6^ bp for 300 mg/kg, respectively. In all laboratories, a clear dose-dependent increase in G:C to T:A mutations was observed, consistent with the known mutation profile of BP [9, 28]. The fold-increases in G:C to T:A mutation frequencies compared to vehicle controls were 3.0, 3.0, and 2.4 (150 mg/kg of BP) and 9.7, 11.0, and 9.0 (300 mg/kg of BP) for labs A, B, and C, respectively. Statistically significant increases in OMFs were observed at 150 and 300 mg/kg in all laboratories (*p <* 0.05). Similarly, the mean OMFs in ENU-exposed samples of the labs A, B and C were comparable: 0.146 × 10^−6^, 0.138 × 10^−6^, and 0.157 × 10^−6^ bp (olive oil), 0.706 × 10^−6^, 0.655 × 10^−6^, and 0.685 × 10^−6^ bp (75 mg/kg), 2.25 × 10^−6^, 2.23 × 10^−6^, and 2.24 × 10^−6^ bp (150 mg/kg), respectively (Fig. 2). In all laboratories, a clear dose-dependent increase in A:T mutations were observed, which was consistent with previous reports on ENU-induced mutations [9, 29]. Statistically significant increases in OMFs were observed at doses of 75 and 150 mg/kg (*p <* 0.05) in all laboratories. The mean frequencies of six types of base substitutions and OMFs in MNU-exposed samples in the labs A and B are shown in Fig. 3. The mean OMFs from labs A and B were 0.258 × 10^−6^ and 0.280 × 10^−6^ bp (12.5 mg/kg) and 0.667 × 10^−6^ and 0.621 × 10^−6^ bp (25 mg/kg), respectively. Statistically significant increases in OMFs were observed at 12.5 and 25 mg/kg in both laboratories (*p <* 0.05). Statistically significant increases in OMFs in Hawk-Seq™ analysis were identified at the same or lower doses compared to those in *gpt* mutant frequencies (MFs). The counts of all mutations and consensus bases in the three laboratories are summarized in Supplementary Table 3.

### Reproducibility of mutation frequencies between laboratories

**Fig. 4:**
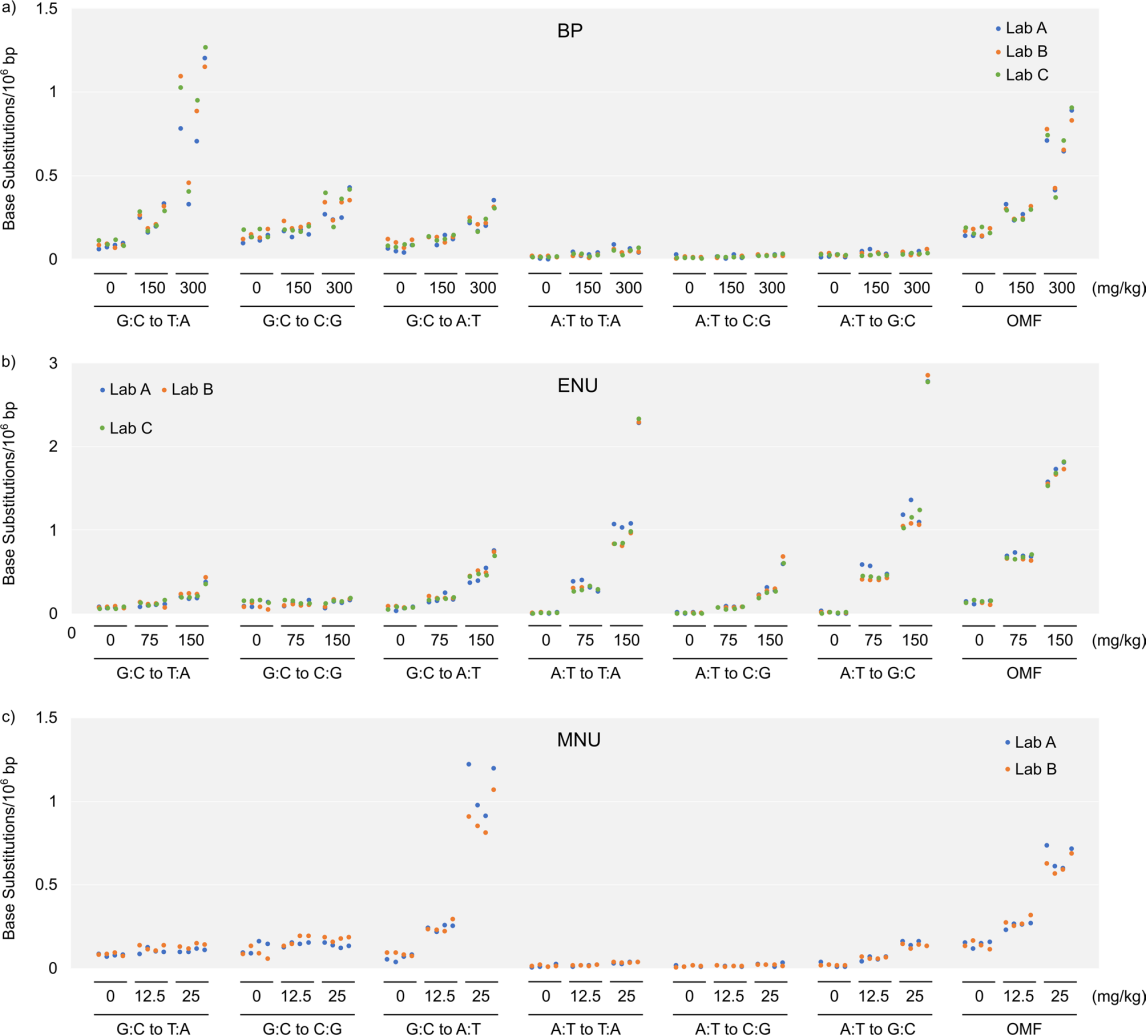
Frequencies of six types of base substitutions and OMFs in individual samples from (**a**) BP-, (**b**) ENU-, and (**c**) MNU-exposed samples. Each plot in blue, orange, and green indicates the value from labs A, B, and C, respectively

**Fig. 5:**
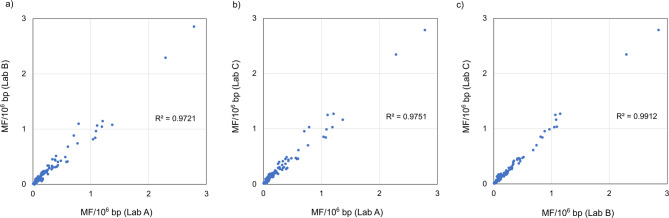
Correlation of the frequencies of six types of base substitutions in BP- and ENU-exposed samples between (**a**) lab A and lab B, (**b**) lab A and lab C, and (**c**) lab B and lab C. The values from the 24 samples (144 plots) are plotted in the graphs

Figure 4a to 4c show the frequencies of six types of BSs and the OMF in individual samples exposed to vehicle control, BP, ENU, and MNU across all laboratories. The mutation spectra and statistical test results among the treatment groups were similar between laboratories, and the MFs were nearly identical, even at the individual animal level. Even within the same dose group (e.g., 300 mg/kg of BP), samples that indicated higher mutation frequencies in lab A’s experiment also indicated higher values in other two laboratories’ experiments. We evaluated the correlation of the six types of BS frequencies between the three laboratories using the data of 24 samples exposed to BP and ENU (144 plots each for lab A-lab B, lab A-lab C, and lab B-lab C). The frequencies of the six types of BS between the three laboratories were highly correlated between laboratories, with r^2^ values of 0.97, 0.98, and 0.99 for lab A-lab B, lab A-lab C, and lab B-lab C, respectively (Fig. 5a to 5c). These data suggest that the between-laboratory reproducibility of the mutation frequencies of the same samples was substantially high for Hawk-Seq™ analysis.

### Comparison of the mutation detection sensitivity between Hawk-Seq™ and ***gpt*** assay

**Fig. 6:**
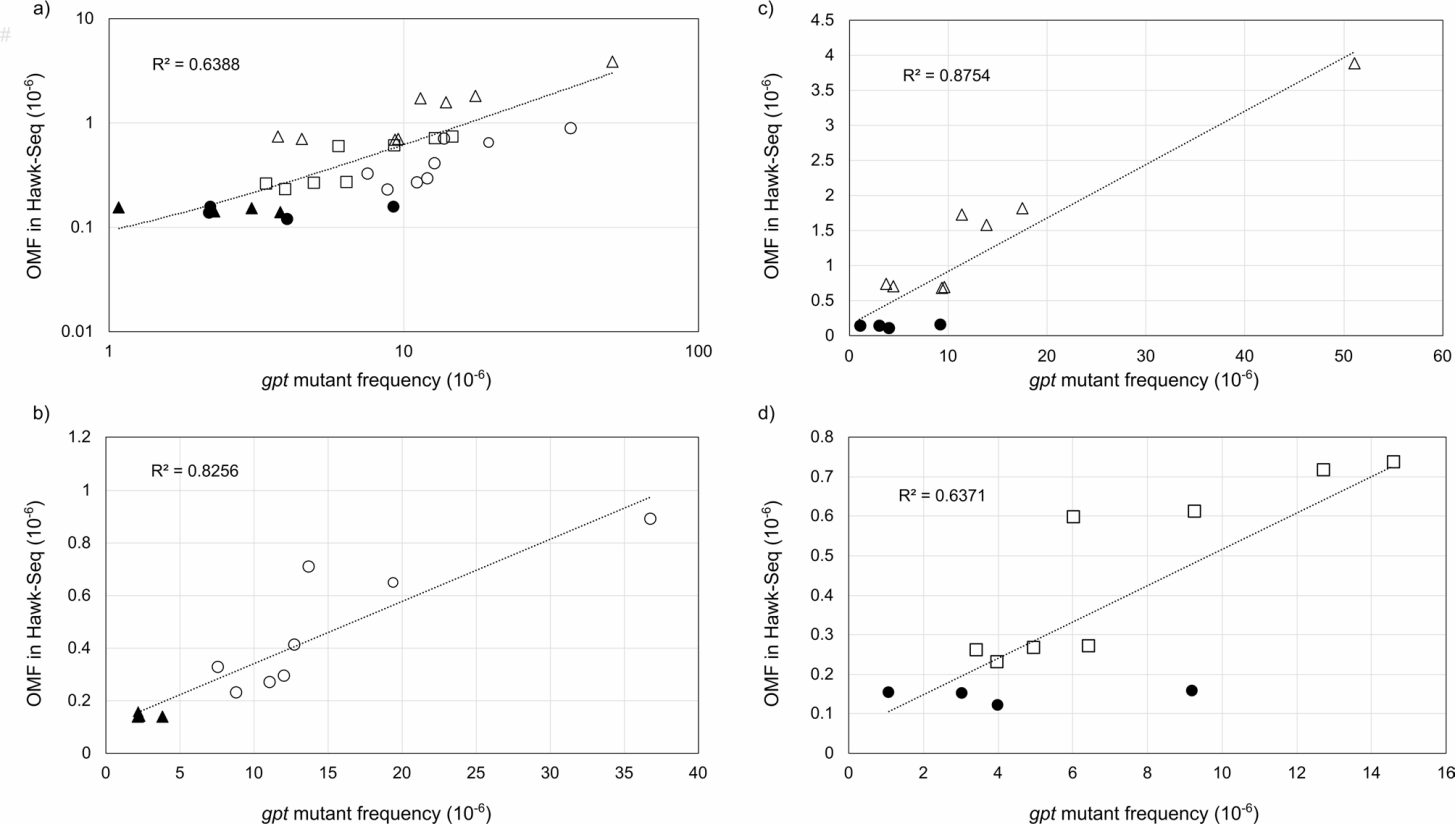
Correlation between OMFs obtained from Hawk-Seq™ analysis (lab A) and *gpt* mutant frequencies. The values of olive oil (black triangle), saline (black circle), ENU (white triangle), MNU (white rectangle), and BP (white circle) exposed samples are shown. The correlations are presented for: **a **all 32 samples, **b **olive oil and BP-exposed samples, **c **saline and ENU-exposed samples, and **d **saline and MNU-exposed samples

We calculated correlations between Hawk-Seq™ OMFs (lab A) and *gpt* MFs. Hawk-Seq™ OMFs and *gpt* MFs indicated positive correlations (Fig. 6). As observed in Fig. 6a the responses may vary depending on the mutagens. Larger responses were observed in Hawk-Seq™ analysis than in the *gpt* assay for ENU- and MNU-exposed samples. In contrast, OMFs in Hawk-Seq™ analysis of BP-exposed samples were generally lower than in the *gpt* mutant frequency. Table 3 summarizes the OMFs and their fold-change values compared to the vehicle controls for Hawk-Seq™ analysis in the three laboratories. Hawk-Seq™ indicated at least 14.2 and 4.5-fold-change values compared to vehicle controls, whereas those were 6.1 and 2.5 on the *gpt* assay for ENU- and MNU-exposed samples, respectively. Meanwhile, the response on BP-exposed samples might be attenuated for Hawk-Seq™ compared to the *gpt* assay; Hawk-Seq™ indicated a 4.6-fold increase at the highest in the three laboratories, whereas an 8.2-fold increase was identified in the *gpt* assay. Similar attenuated responses were also observed in BP-exposed samples on OMFs determined by ecNGS compared to the MFs in TGR assays [14, 21]. These observations suggest that the responses in mutation frequencies determined by ecNGS analysis do not directly correspond to the responses on TGR models. Meanwhile, in the Hawk-Seq™ analysis, statistically significant increases in OMF were identified at the same or lower doses compared to those in *gpt* MFs (Table 3). This is probably because of the relatively lower inter-animal variations in OMFs obtained using Hawk-Seq™ compared to those in *gpt* MFs (Table 1; Fig. 6, and Supplementary Table 4). These data suggest that Hawk-Seq™ enables a sufficiently sensitive mutagenicity test compared with current TGR assays.

**Table 3:** Comparison of fold-change values between OMF from Hawk-Seq™ data and *gpt* assay

Materials		Lab A		Lab B		Lab C		*gpt* assay [9]	
	Dose (mg/kg/day)	OMF (10^−6^) (Mean$\:\pm\:$SD)	Fold increase	OMF (10^−6^) (Mean$\:\pm\:$SD)	Fold increase	OMF (10^−6^) (Mean$\:\pm\:$SD)	Fold increase	Mutant frequency (10^−6^) (Mean$\:\pm\:$SD)	Fold increase
BP	0 (Olive oil)	0.14$\:\pm\:$0.0097	-	0.17$\:\pm\:$0.020	-	0.17$\:\pm\:$0.021	-	2.43$\:\pm\:$0.81	-
	150	0.28$\:\pm\:$0.041 ^S^	2.0	0.28$\:\pm\:$0.038 ^S^	1.6	0.27$\:\pm\:$0.034 ^S^	1.5	9.14$\:\pm\:$2.35 ^S^	3.8
	300	0.67$\:\pm\:$0.20 ^S^	4.6	0.67$\:\pm\:$0.18 ^S^	4.0	0.68$\:\pm\:$0.23 ^S^	3.9	19.91$\:\pm\:$9.79 ^S^	8.2
ENU	0 (Saline)	0.15$\:\pm\:$0.017	-	0.14$\:\pm\:$0.021	-	0.16$\:\pm\:$0.015	-	3.85$\:\pm\:$3.19	-
	75	0.71$\:\pm\:$0.023 ^S^	4.8	0.65$\:\pm\:$0.012 ^S^	4.7	0.68$\:\pm\:$0.022 ^S^	4.4	6.61$\:\pm\:$2.73	1.7
	150	2.3$\:\pm\:$1.1 ^S^	15.4	2.2$\:\pm\:$1.2 ^S^	16.1	2.2$\:\pm\:$1.1 ^S^	14.2	23.48$\:\pm\:$18.58 ^S^	6.1
MNU	12.5	0.26$\:\pm\:$0.019 ^D^	1.8	0.28$\:\pm\:$0.028 ^D^	2.0	NT		4.50$\:\pm\:$1.21	1.2
	25	0.67$\:\pm\:$0.071 ^D^	4.6	0.62$\:\pm\:$0.053 ^D^	4.5	NT		9.60$\:\pm\:$4.03 ^D^	2.5

The data of the *gpt* assay was obtained from a previous study [9]. ENU and MNU share the same vehicle control group. NT; not tested. ^D^: *p* < 0.05 by Dunnett’s test. ^S^: *p* < 0.05 by Steel’s test

## Discussion

We evaluated the sensitivity and inter-laboratory reproducibility of mutation frequencies under Hawk-Seq™ analysis using the same mutagen-exposed DNA samples from *gpt* delta mice. Our data demonstrate that Hawk-Seq™-based analysis has high inter-laboratory reproducibility. Our data also show a strong correlation between the OMF in Hawk-Seq™ analysis and *gpt* mutant frequency. These observations are consistent with those of previous studies using duplex sequencing [21–23]. Therefore, ecNGS-based assays provide mutation data with substantial reproducibility and concordance to TGR assays. Moreover, ecNGS simultaneously provides key additional information on the mutagenic mechanisms associated with the test chemical, which presents a significant advantage over the TGR assay.

Multiple ecNGS methodologies have been developed [1, 2]. These methods differ in terms of molecular barcode usage, DNA fragmentation procedures (sonication or enzymatic digestion), and target genomic regions (panel- or random-based) [3]. Non-target methods over panel-based methods and sonication-based methods over enzyme-based methods allow broader analysis for the entire genome, which allows us to obtain mutation data with a smaller amount of genomic DNA [1, 9, 12, 14, 21]. In our previous study, the mean percentage of the genome covered by at least one consensus base was 25.8 ± 3.9% (*n* = 12) in Hawk-Seq™ analysis [9], which provided mutation profiles across a large genomic region. In contrast, panel-based methods can more precisely distinguish single mutations from clonal or subclonal mutations, which enables analysis with lower background errors regardless of sample heterogeneity [1, 14, 21]. Therefore, for evaluating mutagenicity across various biological models, having multiple ecNGS methods available as alternatives is preferable. Our data suggests that our method could be one of the ecNGS candidates to enable robust analysis of mutagen-induced mutations.

For genome-scale approaches such as Hawk-Seq™, distinguishing germline mutations (noise) from somatic mutations (target) can be challenging. One approach to minimizing the effect of germline mutations is to conduct whole-genome sequencing of individual animals; however, this would double the sequencing cost for mutation analysis. However, as shown in this study, our approach using SNP information from public databases [26] and internal variant information from representative animals (Supplementary Fig. 2 and Supplementary Table 2) is sufficient to achieve high sensitivity, at least for the *gpt* delta mouse, an inbred strain derived from the C57BL/6J background [30, 31]. This approach could support cost-effective genome-scale ecNGS analysis. In the future, sharing genetic variant information for widely used rodent strains within the genotoxicity research community would help increase detection sensitivity and reduce associated cost.

Hawk-Seq™ demonstrated high inter-laboratory reproducibility. The correlation coefficient values (r^2^ = 0.972–0.991) were comparable to those reported in previous duplex sequencing studies using mouse (r^2^ = 0.94) [21], rat (*r* = 0.986) [22], and TK6 (*r* = 0.97) [18] samples. The coefficient of variation (CV) for OMFs between laboratories (inter-laboratory variation for the same samples) ranged from 0.0072 to 0.230 (Supplementary Table 4). In contrast, CV values for OMFs between animals (inter-animal variation within the same dose group) ranged from 0.0189 to 0.521. These findings suggest that inter-laboratory variation is smaller than inter-animal variation in Hawk-Seq™ analysis. Therefore, Hawk-Seq™-based analysis appears to have sufficient reproducibility to yield consistent results across laboratories under the protocol of a TGR model. 

OMFs obtained using Hawk-Seq™ analysis and the MFs in the *gpt* assay of individual animals also indicated good positive correlations (Supplementary Fig. 3). This suggests that Hawk-Seq™ can function as an alternative method for OECD TG488, as previously proposed for ecNGS in a previous review [1]. However, regarding BP-exposed samples, although Hawk-Seq™ identified a clear increase in BP-induced G:C to T:A mutations, the mutant frequency on the *gpt* assay was higher than the OMFs on Hawk-Seq™ analysis. Regarding BP, similar trends were also observed in studies using mice, in which the mutant frequency in TGR models indicated higher fold-inductions compared to those in duplex sequencing [14, 21]. These observations suggest that the sensitivity of ecNGS analysis compared to TGR gene mutation assays may vary depending on the mutagens used. However, this may be a BP-specific observation because previous studies have indicated that exogenous bacterial genes are a preferential target for BP mutagenesis [32, 33]. Additionally, the G:C content and the sequence context (e.g. trinucleotide context) may influence whether a mutagen produces a stronger or weaker response in a representative genomic locus. Therefore, the evaluation of mutagens of various mechanisms by ecNGS and understanding their relationship with mutation distribution would be useful for fully interpreting the results of ecNGS analyses.

Alongside the promotion of ecNGS-based assays, it is essential to update our understanding of the relationship between mutagenicity and carcinogenicity. For example, further discussion within the expert community is needed to determine how to interpret specific findings, such as significant increases in a particular mutation subtype or in a specific genomic region. To correctly interpret these outcomes and evaluate safety risks, the accumulation of ecNGS-based data is required. Therefore, ecNGS methodologies should be more widely adopted in the field of genotoxicity research.

## Conclusions

Hawk-Seq™ demonstrated strong inter-laboratory reproducibility and concordance with *gpt* assay results in this study on three mutagens. Thus, our study provides additional evidence to support that ecNGS methods provide strong alternative method to TGR gene mutation assays.

## Supplementary Information

Supplementary Material 1.

Supplementary Material 2.

## Data Availability

Data will be provided upon request.
