# Supplementary material for: Whole genome mutagenicity evaluation using Hawk-Seq™ demonstrates high inter-laboratory reproducibility and concordance with the transgenic rodent gene mutation assay
Source: Genes Environ. 2025 Jul 29;47:13. doi: 10.1186/s41021-025-00336-w (PMC12305950; doi:10.1186/s41021-025-00336-w)
Supplement: Supplementary file 1 — Supplementary Material 1. [file 41021_2025_336_MOESM1_ESM.pdf]

Whole genome mutagenicity evaluation using Hawk-Seq™ demonstrates high inter-laboratory reproducibility and concordance with the transgenic rodent gene mutation assay

Shoji Matsumura[1]*, Sayaka Hosoi[1], Takako Hirose[1], Yuki Otsubo[1], Kazutoshi Saito[2], Masaaki Miyazawa[2], Akihiro Kawade[3], Atsushi Hakura[3], Dai Kakiuchi[4], Shoji Asakura[4], Naoki Koyama[5],Yuki Okada[6], Satsuki Chikura[6], Takafumi Kimoto[6], Kenichi Masumura[7], Takayoshi Suzuki[8], Kei-ichi Sugiyama[8]

[1]R&D -Safety Science Research, Kao Corporation, 3-25-14 Tonomachi, Kawasaki-ku, Kawasaki-shi, Kanagawa 210-0821, Japan
[2]R&D -Safety Science Research, Kao Corporation, 2606 Akabane, Ichikai-Machi, Haga-Gun, Tochigi 321-3497, Japan
[3] Drug Safety & Animal Care Technology Unit, Tsukuba Division, Sunplanet Co., Ltd., 5-1-3 Tokodai, Tsukuba-shi, Ibaraki 300-2635, Japan
[4]Global Drug Safety, Eisai Co., Ltd., 5-1-3 Tokodai, Tsukuba-shi, Ibaraki 300-2635, Japan
[5]Translational Research Division, Safety and Bioscience Research Dept., Chugai Pharmaceutical Co., Ltd., 216 Totsuka, Totsuka-ku, Yokohama-shi, Kanagawa, 244-8602, Japan
[6]Teijin Pharma Limited, 4-3-2 Asahigaoka, Hino, Tokyo 191-8512, Japan
[7]Division of Risk Assessment, National Institute of Health Sciences, 3-25-26 Tonomachi, Kawasaki-ku, Kawasaki-shi, Kanagawa, 210-9501, Japan
[8]Division of Genome Safety Science, National Institute of Health Sciences, 3-25-26 Tonomachi, Kawasaki-ku, Kawasaki-shi, Kanagawa, 210-9501, Japan

*To whom correspondence should be addressed. Tel: +81-70-3301-1852; Fax; +81-285-68-7452; Email: matsumura.shouji@kao.com

Supplementary data

a)

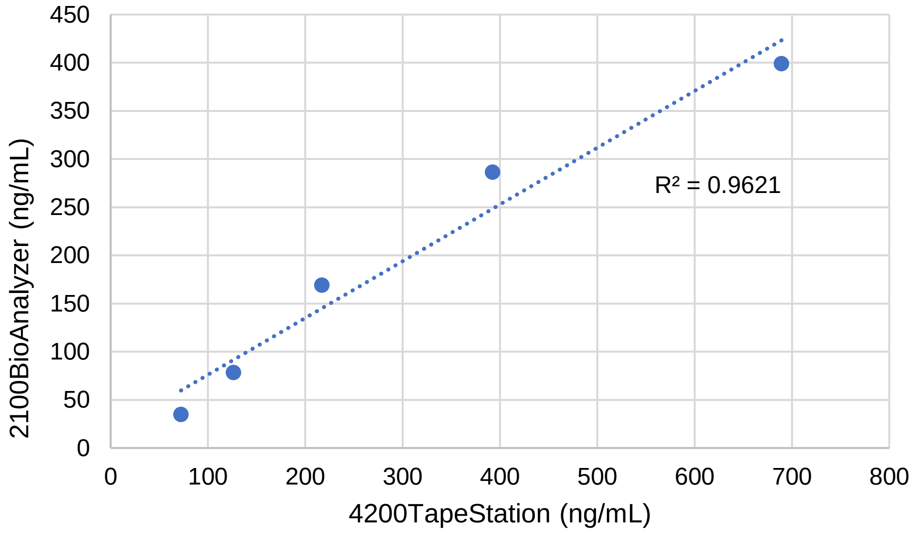

450
400
350
300
250
200
150
100
50
0
0
100
200
300
400
500
600
700
800
2100BioAnalyzer (ng/mL)
4200TapeStation (ng/mL)
R² = 0.9621

b)

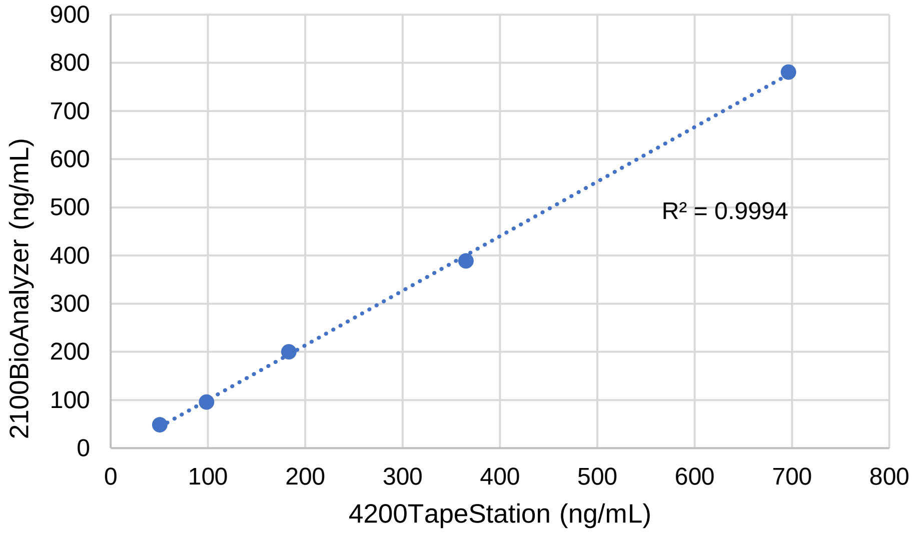

900
800
700
600
500
400
300
200
100
0
0
100
200
300
400
500
600
700
800
2100BioAnalyzer (ng/mL)
4200TapeStation (ng/mL)
R² = 0.9994

c)

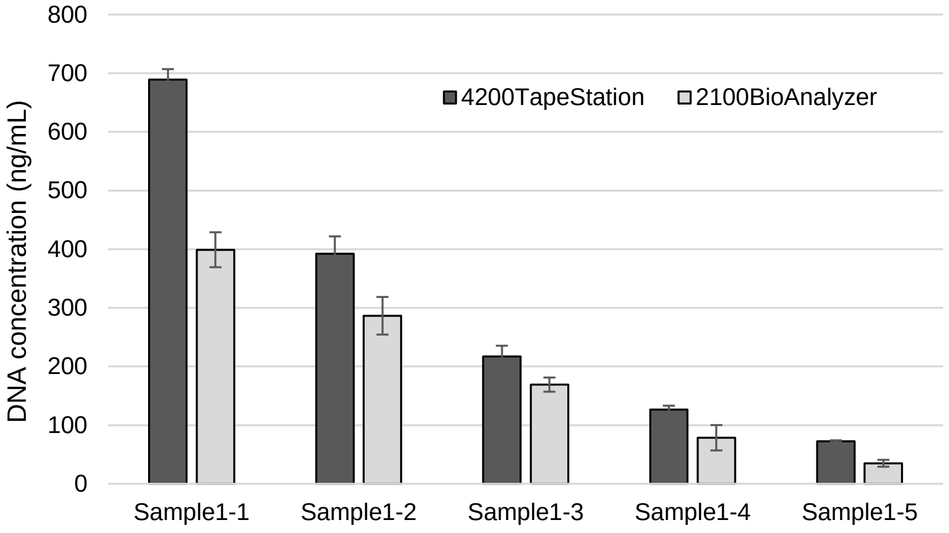

d)

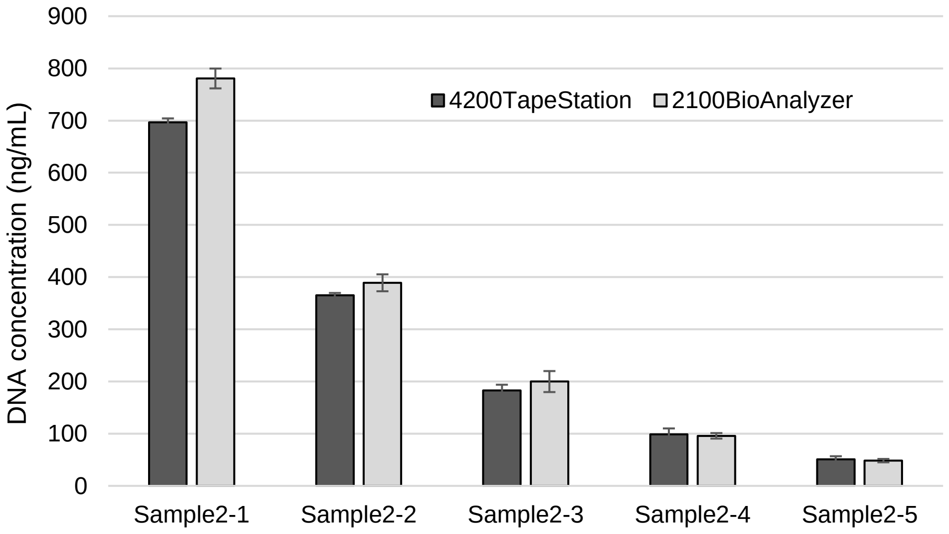

Supplementary Fig. 1 Comparison between the 4200 TapeStation and 2100 BioAnalyzer for determining the concentrations of the adapter-ligated products and PCR-enriched products obtained during library preparation. Using a mouse DNA sample (spleen from animal ID. 3002), DNA samples adjusted to five concentrations with two-fold serial dilutions were prepared both after adapter ligation (Sample1-1 to 1-5) and PCR enrichment (Sample2-1 to 2-5). The concentrations of these samples were measured thrice for each sample using two instruments. The correlations of the mean values from the two instruments for a) the adapter-ligated products and b) PCR-enriched products are shown. The

mean concentrations with standard deviations from the two instruments for solutions of c) the adapter-ligated products, and d) PCR-enriched products after being appropriately diluted are shown. For the adapter-ligated products, 2100BioAnalyzer indicated lower values than those of 4200TapeStation, especially at relatively higher concentrations. Therefore, the same instrument should be used throughout the mutagenicity tests during the optimization of PCR conditions to maximize dsDCS yields. If optimal conditions are determined using the same instrument, differences in concentration values do not significantly affect experimental outcomes because both instruments demonstrated substantial reproducibility of DNA concentrations.

a)

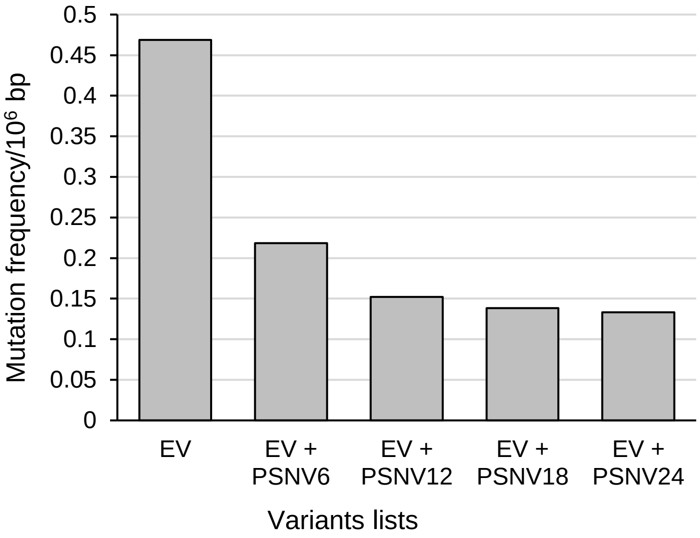

b)

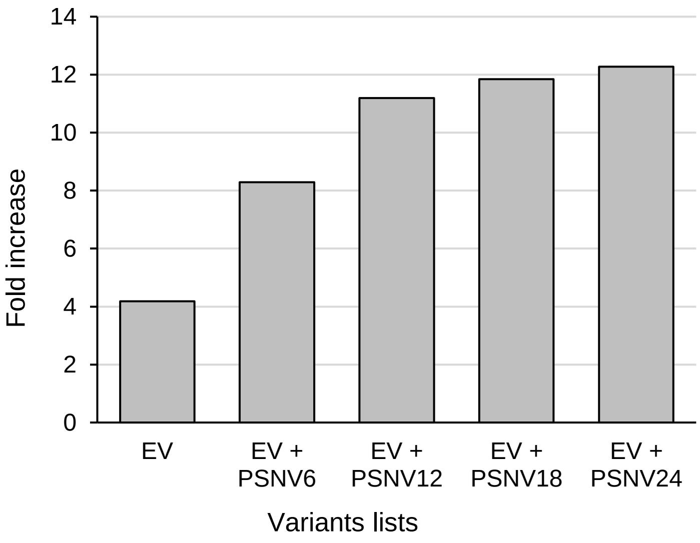

Supplementary Fig. 2 Effect of filtering out possible variant positions from the mutation analysis on the background error frequency and mutation detection sensitivity. The genomic positions that possibly harbored SNPs were listed using mutation data from samples exposed to vehicle controls, ENU, and BP (Supplementary Table 2). Specifically, the positions at which mutations were detected in two or more samples were listed as possible SNP (PSNP) positions. PSNP lists PSNP6, PSNP12, PSNP18, and PSNP24 were created using data from 6, 12, 18, and 24 samples, respectively (Supplementary Table 2). a) The overall mutation frequency in the control sample (animal ID. 3001)

under the analysis using Ensembl Variations release 102 (EV) and EV + each PSNP list as filtering targets. The overall mutation frequency decreased according to the number of samples used to create the PSNP lists; it decreased to the order of 10^{-7} bp when 12 or more samples were used to create the PSNP list. b) The fold change values of overall mutation frequencies in a sample exposed to 150 mg/kg of ENU (mean of 3302 and 6301) compared to that of the control (mean of 3001 and 6001). The fold increase values increased with the number of samples used for PSNP preparation.

Supplementary Table 1 Summary of the efficiency of generating dsDCS read pairs depending on the input DNA amount for PCR enrichment.

	DNA input amount (amol)	No. of read pairs sequenced ($\times 10^6$)	No. of read pairs after filtering by Cutadapt ($\times 10^6$)	No. of dsDCS read pairs($\times 10^6$)	No. of dsDCS read pairs/No. of read pairs sequenced (%)	No. of dsDCS read pairs/No. of read pairs after filtering by Cutadapt (%)
Lab B	19.5	101.5	68.6	0.985	0.970	1.44
	39	106.1	69.5	1.91	1.80	2.75
	78	64.1	41.5	2.47	3.85	5.95
	156	86.0	51.3	4.63	5.38	9.03
	312	81.2	52.4	3.20	3.94	6.10
	624	96.1	63.3	2.64	2.75	4.17
Lab C	19.5	121.5	84.1	1.75	1.44	2.08
	39	91.3	58.4	3.69	4.04	6.32
	78	108.7	74.4	5.58	5.13	7.49
	156	125.0	82.3	7.35	5.88	8.93
	312	136.1	90.0	6.18	4.54	6.87
	624	123.9	84.8	3.95	3.19	4.66

Supplementary Table 2 Sample list for creating each PSNP list.

Mutagens	Animal ID.	EV	No. of samples used to make PSNP list			
			6	12	18	24
Saline (vehicle)	3001		X	X	X	X
	6001		X	X	X	X
	6002		X	X	X	X
	6003		X	X	X	X
Olive oil	1002		X	X	X	X
	1003		X	X	X	X
	1004			X	X	X
	1005			X	X	X
ENU_75	3201			X	X	X
	3202			X	X	X
	6201			X	X	X
	6203			X	X	X
ENU_150	3302				X	X
	6301				X	X
	6302				X	X
	6303				X	X
BP_150	1201				X	X
	1203				X	X
	1204					X
	1205					X
BP_300	1301					X
	1302					X
	1303					X
	1305					X
EV		X	X	X	X	X

X: Samples used for EV and PSNP lists

Supplementary Table 3 No. of total consensus bases and mutations in each sample in each laboratory

		Lab A		Lab B		Lab C	
Materials	Dose (mg/kg/day)	Consensus bases	Mutations	Consensus bases	Mutations	Consensus bases	Mutations
BP	0 (Olive oil)	972,244,248	136	432,293,852	73	2,076,694,502	393
		731,745,299	103	1,569,402,519	283	1,858,739,945	286
		884,265,347	122	1,100,710,090	155	1,352,966,718	264
		963,746,525	153	632,455,640	117	1,584,417,181	249
	150	915,708,042	302	572,465,538	172	2,304,862,944	681
		892,235,392	208	825,703,738	200	1,756,073,019	417
		1,058,855,192	287	641,738,598	157	1,814,826,902	437
		861,359,784	256	973,968,627	309	1,427,059,931	429
	300	862,027,968	613	1,209,268,298	941	2,965,130,837	2,204
		761,249,708	316	1,022,896,326	434	1,758,312,954	655
		971,223,483	629	1,403,559,722	918	1,771,290,183	1,263
		675,930,806	603	556,147,552	463	1,844,711,365	1,678
ENU	0 (Saline)	976,600,772	150	674,948,645	91	2,162,659,726	295
		852,895,515	103	547,967,577	91	1,436,907,144	249
		945,383,640	144	696,463,417	96	1,938,796,867	309
		838,127,525	133	892,499,682	102	1,843,886,489	295
	75	877,369,110	614	785,969,236	526	1,437,193,331	969
		864,923,830	639	1,010,586,484	661	1,458,547,663	970

		661,001,361	461	988,981,067	649	1,889,690,186	1,292
		987,679,276	678	613,415,509	392	1,621,074,000	1,161
	150	962,347,849	1,521	409,367,141	637	1,496,152,888	2,298
		865,327,781	1,498	1,065,579,149	1,775	1,834,256,943	3,098
		802,992,170	1,458	519,354,960	901	1,656,726,369	3,025
		1,052,892,333	4,085	799,839,830	3,179	2,186,776,985	8,507
MNU	12.5	966,388,117	223	839,358,946	231		
		927,399,277	249	1,131,141,557	289	NT	
		1,100,855,030	289	1,186,449,166	318		
		897,966,642	244	1,370,862,151	439		
	25	712,727,264	526	892,713,707	563		
		976,528,048	599	823,763,140	468	NT	
		866,220,538	519	841,361,867	499		
		1,015,215,044	728	713,819,693	493		

Supplementary Table 4 Summary of the inter-laboratory variations and the inter-animal variations in the OMFs (×10^{-6} bp).

	Animal ID								Animal ID						
Olive oil	1002	1003	1004	1005	Mean	SD	CV	ENU 75 mg/kg	3201	3202	6201	6203	Mean	SD	CV
Lab A	0.140	0.141	0.138	0.159	0.144	0.010	0.067	Lab A	0.700	0.739	0.697	0.686	0.706	0.023	0.032
Lab B	0.169	0.180	0.141	0.185	0.169	0.020	0.117	Lab B	0.669	0.654	0.656	0.639	0.655	0.012	0.019
Lab C	0.189	0.154	0.195	0.157	0.174	0.021	0.123	Lab C	0.674	0.665	0.684	0.716	0.685	0.022	0.033
Mean	0.166	0.158	0.158	0.167				Mean	0.681	0.686	0.679	0.681			
SD	0.025	0.020	0.032	0.016				SD	0.016	0.046	0.021	0.039			
CV	0.149	0.127	0.204	0.094				CV	0.024	0.067	0.031	0.057			

	Animal ID								Animal ID						
Saline	3001	6001	6002	6003	Mean	SD	CV	ENU 150 mg/kg	3302	6301	6302	6303	Mean	SD	CV
Lab A	0.154	0.121	0.152	0.159	0.146	0.017	0.118	Lab A	1.58	1.73	1.82	3.88	2.25	1.09	0.48
Lab B	0.135	0.166	0.138	0.114	0.138	0.021	0.154	Lab B	1.56	1.67	1.73	3.97	2.23	1.16	0.52
Lab C	0.136	0.173	0.159	0.160	0.157	0.015	0.097	Lab C	1.54	1.69	1.83	3.89	2.24	1.11	0.50
Mean	0.142	0.153	0.150	0.144				Mean	1.56	1.70	1.79	3.91			
SD	0.010	0.028	0.011	0.026				SD	0.02	0.03	0.05	0.05			
CV	0.074	0.186	0.073	0.180				CV	0.01	0.02	0.03	0.01			

	Animal ID								Animal ID						
BP	1201	1203	1204	1205	Mean	SD	CV	MNU	5101	5102	8101	8102	Mean	SD	CV
150 mg/kg								12.5 mg/kg							
Lab A	0.330	0.233	0.271	0.297	0.283	0.041	0.145	Lab A	0.231	0.268	0.263	0.272	0.258	0.019	0.073
Lab B	0.300	0.242	0.245	0.317	0.276	0.038	0.139	Lab B	0.275	0.255	0.268	0.320	0.280	0.028	0.101
Lab C	0.295	0.237	0.241	0.301	0.269	0.034	0.127	Mean	0.253	0.262	0.265	0.296			
Mean	0.309	0.238	0.252	0.305				SD	0.031	0.009	0.004	0.034			
SD	0.019	0.005	0.016	0.011				CV	0.124	0.035	0.015	0.116			
CV	0.060	0.019	0.065	0.035											

	Animal ID								Animal ID						
BP	1301	1302	1303	1305	Mean	SD	CV	MNU	5202	8201	8202	8203	Mean	SD	CV
300 mg/kg								25 mg/kg							
Lab A	0.711	0.415	0.648	0.892	0.666	0.197	0.296	Lab A	0.738	0.613	0.599	0.717	0.667	0.071	0.106
Lab B	0.778	0.424	0.654	0.833	0.672	0.181	0.270	Lab B	0.631	0.568	0.593	0.691	0.621	0.053	0.086
Lab C	0.743	0.373	0.713	0.910	0.685	0.225	0.329	Mean	0.684	0.591	0.596	0.704			
Mean	0.744	0.404	0.672	0.878				SD	0.076	0.032	0.004	0.019			
SD	0.034	0.028	0.036	0.040				CV	0.111	0.054	0.007	0.027			
CV	0.045	0.068	0.054	0.046											
