# Supplementary material for: Whole genome mutagenicity evaluation using Hawk-Seq™ demonstrates high inter-laboratory reproducibility and concordance with the transgenic rodent gene mutation assay
Source: Genes Environ. 2025 Jul 29;47:13. doi: 10.1186/s41021-025-00336-w (PMC12305950; doi:10.1186/s41021-025-00336-w)
Supplement: Supplementary file 2 — Supplementary Material 2. [file 41021_2025_336_MOESM2_ESM.pdf]

Whole genome mutagenicity evaluation using Hawk-Seq™ demonstrates high inter-laboratory reproducibility and concordance with the transgenic rodent gene mutation assay

Shoji Matsumura[1]*, Sayaka Hosoi[1], Takako Hirose[1], Yuki Otsubo[1], Kazutoshi Saito[2], Masaaki Miyazawa[2], Akihiro Kawade[3], Atsushi Hakura[3], Dai Kakiuchi[4], Shoji Asakura[4], Naoki Koyama[5], Yuki Okada[6], Satsuki Chikura[6], Takafumi Kimoto[6], Kenichi Masumura[7], Takayoshi Suzuki[8], Kei-ichi Sugiyama[8]

[1]R&D -Safety Science Research, Kao Corporation, 3-25-14 Tonomachi, Kawasaki-ku, Kawasaki-shi, Kanagawa 210-0821, Japan

[2]R&D -Safety Science Research, Kao Corporation, 2606 Akabane, Ichikai-Machi, Haga-Gun, Tochigi 321-3497, Japan

[3] Drug Safety & Animal Care Technology Unit, Tsukuba Division, Sunplanet Co., Ltd., 5-1-3 Tokodai, Tsukuba-shi, Ibaraki 300-2635, Japan

[4]Global Drug Safety, Eisai Co., Ltd., 5-1-3 Tokodai, Tsukuba-shi, Ibaraki 300-2635, Japan

[5]Translational Research Division, Safety and Bioscience Research Dept., Chugai Pharmaceutical Co., Ltd., 216 Totsuka, Totsuka-ku, Yokohama-shi, Kanagawa, 244-8602, Japan

[6]Teijin Pharma Limited, 4-3-2 Asahigaoka, Hino, Tokyo 191-8512, Japan

[7]Division of Risk Assessment, National Institute of Health Sciences, 3-25-26 Tonomachi, Kawasaki-ku, Kawasaki-shi, Kanagawa, 210-9501, Japan

[8]Division of Genome Safety Science, National Institute of Health Sciences, 3-25-26 Tonomachi, Kawasaki-ku, Kawasaki-shi, Kanagawa, 210-9501, Japan

*To whom correspondence should be addressed. Tel: +81-70-3301-1852; Fax; +81-285-68-7452; Email: matsumura.shouji@kao.com

Supplementary materials and methods

Detailed library preparation protocol (Ver. 2025.05)

Library preparation for Hawk-Seq™ was conducted according to the protocol of the TruSeq library prep kit (Illumina, San Diego, CA, USA). The conditions were modified to optimize the Hawk-Seq™ analysis. The detailed protocol presented here describes a typical workflow and the know-how to efficiently perform the experiment.

Outline of the overall experimental workflow.

1. Quantification of the genomic DNA (gDNA) concentration using Qubit
2. Electrophoresis of gDNA samples by 4200TapeStation (Optional)
3. Fragmentation of gDNA samples
4. End repair and size selection
5. Adenylation of 3′ ends of DNA fragment
6. Adapter ligation
7. Enrichment of DNA fragment by PCR
8. Pooling of the library DNA

A detailed description of each experimental step is provided.

1. Quantification of the genomic DNA (gDNA) concentration using Qubit

- Reagents and Setups

- gDNA: Thaw at room temperature (RT) and mix well by pipetting.
- Qubit 3.0 Fluorometer (Qubit Flex Fluorometer is also applicable) (Thermo Fisher Scientific, MA, USA).
- Qubit assay tubes (not compatible with Qubit Flex Fluorometer).
- Qubit dsDNA BR Assay Kit.
- Qubit dsDNA BR Reagent: Warm to RT before use.
- Qubit dsDNA BR buffer: Store at RT.
- Qubit dsDNA BR standards 1 and 2: Warm to RT before use.

- Protocols

- The working solution was prepared by mixing Qubit dsDNA BR Reagent and Qubit dsDNA BR buffer as follows: (Reagent 1 µL + buffer 199 µL) × (No. of gDNA samples + 1 extra + 2 standards).
- Mix 190 µL of the working solution and 10 µL of each standard. Transfer them to Qubit assay tubes (standard diluents).
- Mix 199 µL of working solution and 1 µL of gDNA samples. Transfer them to Qubit assay tubes

(gDNA diluents).

- Incubate each diluent at RT for 2 min.
- Set two standard diluents to the Qubit and determine the calibration curve.
- Set each gDNA diluents to Qubit and measure each gDNA sample concentration.

2. Electrophoresis of gDNA samples by 4200TapeStation (Optional)

- Reagents and Setups

- 4200TapeStation (Agilent Technologies, CA, USA).
- gDNA samples: Thaw at RT and mix well.
- Genomic DNA ScreenTape: Warm to RT before use.
- Genomic DNA reagent kit (marker and sample buffer): Warm to RT before use, then mix well.

- Protocols

- Add 10 µL of sample buffer to a 96 well plate for TapeStation.
- Add 1 µL of gDNA to each well, then mix well using a 10 µL pipet or plate shaker.
- Set a 96-well plate to the TapeStation and perform electrophoresis.
- Confirm the integrity of the gDNA samples.

3. Fragmentation of gDNA samples

- Reagents and Setups

- gDNA samples: Thaw at RT and mix well by pipetting. Minimize the number of freeze-thaw cycles to avoid gDNA deterioration.
- Resuspension Buffer (RSB) in the TruSeq library prep kit: Thaw at RT and mix well. Store at 4 °C after use.
- Sample Purification Beads (SPB) in TruSeq library prep kit: Warm to RT before use.
- 80% EtOH: freshly prepare just before use (99.5% EtOH: deionized water [DW] = 8: 2).
- TE (10 mM Tris-HCl, 1mM EDTA, pH 8.0).
- Covaris ME220 (Covaris, MA, USA).
- 8 micro TUBE-50 AFA Fiber Strip V2 for Covaris ME220.
- Add enough DW to Covaris bottle and set to ME220. Create the program below in advance.
- ME220 Setting.
  - Duty Factor 20%
  - Intensity -
  - Peak/Displayed Power 50W

- Cycles / Burst 1000
- Duration 45
- Mode -
- Temperature 9

- Reagents for 4200 TapeStation
  - High-Sensitivity D1000 (HSD1000) ScreenTape: Warm to RT before use.
  - HSD1000 reagents (marker and sample buffer): Warm to RT before use and mix well.

● Protocols

➢ Fragmentation

- Add 60 ng of each gDNA sample to TE (do not use RSB in TruSeq kit) to prepare 52 μL of gDNA solution. Mix well by pipetting.
- Add 52 μL of gDNA solutions to Covaris tubes (50 + 2 μL to securely obtain 50 μL after fragmentation).
- Fragment gDNA using Covaris under the settings described above (mean fragment size: ca. 350 bp).
- Transfer 50 μL of the supernatant of each sample to a PCR tube.

➢ Clean up of DNA fragment

- Mix the SPB well by turning the tube upside down and vortexing (ensure that it is well-dispersed).
- Add 80 μL of SPB to each supernatant. Mix well by pipetting. Incubate for 5 min at RT.
- Place the samples on the magnetic stand. Incubate for 5 min at RT until the liquid is clear. Remove the supernatant.
- Add 200 μL of 80% EtOH. Incubate for 30 s at RT. Remove the supernatant.
- Repeat this washing process once again on the magnetic stand.
- Remove the supernatant from the bottom of the tube using a 10 μL pipette. Incubate for 5 min at RT on the magnetic stand (sufficiently dry).
- Add 64 μL (TapeStation) or 63 μL (BioAnalyzer) of RSB to each tube. Take each tube from a magnetic stand and mix well by pipetting. Incubate for 2 min at RT.
- Place the samples on the magnetic stand. Incubate for 5 min at RT until the liquid is clear.
- Transfer 62 μL (TapeStation) or 61 μL (BioAnalyzer) of the supernatant of each sample to a new PCR tube.

➢ Electrophoresis (No. 1)

- Conduct electrophoresis using TapeStation (optionally BioAnalyzer) and check the concentration and fragment size of each DNA sample. If any samples indicate anomalous sizes or concentrations, conduct the electrophoresis again or check if the experimental procedures were conducted appropriately.
- Take the 2 μL portion from the supernatant of each sample and mix well with 2 μL of HSD1000 sample buffer (be careful not to include bubbles).
- Subject each mixture to electrophoresis using a TapeStation HSD1000 ScreenTape.

The remaining amount of supernatant of each sample is 60 μL.

4. End repair and size selection

- Reagents and Setups

- End Repair Mix 2 (ER) in the TruSeq library prep kit: Thaw at RT and then place on ice. Store at -20 °C after use.
- RSB: warm to RT.
- SPB in TruSeq library prep kit: warm to RT before use. Ensure that it is well-dispersed.
- 80% EtOH: freshly prepare just before use (99.5% EtOH:DW = 8:2).
- DW.
- Establish the thermal cycler program for ER below.
  - Set the option of pre-heat lid at 100 °C
  - 30 °C for 30 min
  - Hold at 4 °C

- Protocols

- Dispense precise amounts of liquid during the experiment. Slight differences in the bead amount and DNA solutions will substantially affect fragment size to be recovered.

➢ End repair

- Mix the ER well by turning the tube upside down several times.
- Set the tube containing supernatant of each sample on the aluminum block placed on ice. Add 40 μL of ER to each supernatant. Mix well by pipetting (the liquid amount is 60 μL + 40 μL = 100 μL).
- Collect the liquid to the bottom using a benchtop centrifuge. Remove air bubble if observed.
- Set the sample tubes to the thermal cycler and run ER program above (set the liquid amount to 100 μL).

- After the program finished, collect the solutions at the bottom using a benchtop centrifuge.

➢ Removal of large DNA fragment

- Mix the SPB well by turning the tube upside down and vortexing (ensure that it is well-dispersed).
- Dilute the SPB using DW, as described below. Mix well by vortex (SPB diluent).
  (109.25 μL of SPB + 74.75 μL of DW) × No. of samples (amount including 15% of surplus)
- Add 160 μL of SPB diluent to each sample. Mix well by pipetting. Incubate for 5 min at RT. (The liquid amount is 260 μL. Use a PCR tube but ensure that it does not overflow.)
- Place the sample tubes on a magnetic stand. Incubate for 5 min at RT until the liquid is clear.
- Transfer 250 μL of supernatant from each sample to the new PCR tube.

➢ Removal of small DNA fragment

- Mix the SPB well by turning the tube upside down and vortexing (ensure that it is well-dispersed).
- Add 30 μL of SPB to each sample. Mix well by pipetting. Incubate at RT for 5 min. (the liquid amount is 250 μL + 30 μL = 280 μL. Ensure that it does not overflow.)
- Place the sample on the magnetic stand. Incubate for 5 min at RT until the liquid is clear. Remove the supernatant.
- Add 200 μL of 80% EtOH to each sample. Incubate for 30 s at RT. Remove the supernatant.
- Repeat this washing process once again on the magnetic stand.
- Remove the supernatant from the bottom of the tube using 10 μL of pipet. Incubate for 5 min at RT on the magnetic stand (sufficiently dry).
- Add 21.5 μL (20.5 μL if BioAnalyzer is used for electrophoresis below) of RSB to each sample tube. Take each sample tube from a magnetic stand and mix well by pipetting. Incubate for 2 min at RT.
- Place each sample tube on the magnetic stand. Incubate for 5 min at RT until the liquid is clear.
- Transfer 19.5 μL (18.5 μL if BioAnalyzer is used for electrophoresis below) of supernatant from each sample to the new PCR tube with attached cap.

➢ Electrophoresis (No. 2)

- Conduct electrophoresis using TapeStation (optionally BioAnalyzer) and check the concentration and fragment size of each sample. If any samples indicate anomalous sizes or concentrations, conduct the electrophoresis again or check if the experimental procedures were conducted appropriately.
- Take the 2 μL portion from the supernatant of each sample and mix well with 2 μL of HSD1000 sample buffer (be careful not to include air bubbles).
- Subject each mixture to electrophoresis using TapeStation HSD1000 ScreenTape.

The remaining amount of supernatant of each sample is 17.5 μL.
To stop the experiment here temporarily (up to 7 days), store samples at -20 °C.

5. Adenylation of 3′ ends of DNA fragment

- Reagents and Setups

- A-Tailing Mix (ATL) in TruSeq library prep kit: Thaw at RT. Store at - 20°C after use.
- RSB: warm to RT.
- Setup thermal cycler program for ATAIL below.
  - Set the option of pre-heat lid at 100 °C
  - 37 °C for 30 min
  - 70 °C for 5 min
  - 4 °C for 5 min hold at 4 °C

- Protocols

- Mix the ATL well by turning the tube upside down several times.
- Set the tube containing supernatant of each sample on the aluminum block placed on ice. Add 12.5 μL of ATL to each sample. Mix well by pipetting (the liquid amount is 17.5 μL + 12.5 μL = 30 μL).
- Collect the liquid at the bottom using a benchtop centrifuge. Remove air bubbles if observed.
- Set the sample tubes on the thermal cycler and run the ATAIL program above (set the liquid amount to 30 μL).
- After the program finishes, collect the solutions at the bottom using a benchtop centrifuge.

6. Adapter ligation

- Reagents and Setup

- Index Adapter (UD index, 96-well plate): Thaw at RT. Store at -20 °C after use. Limit the freeze and thaw cycle up to three times and use an index from a new well in each experiment if possible.
- Ligation Mix 2 (LIG2) in TruSeq library prep kit: Take out from storage at -20 °C and mix well just before use. Store at - 20°C after use.
- RSB: warm to RT.
- Stop Ligation Buffer (STL) in TruSeq library prep kit: Thaw at RT, then mix well. Store at -20 °C after use.
- SPB: warm to RT.
- 80% EtOH: freshly prepare just before use (99.5% EtOH: DW = 8: 2).

- Reagents for 4200 TapeStation
  - ScreenTape High-Sensitivity D5000 (HSD5000): warm to RT. Store at 4 °C after use. (Be careful not to use HSD1000)
  - HSD5000 reagents (marker and sample buffer): warm to RT, then mix well. Store at 4 °C after use.

- Setup thermal cycler program for LIG below.
  - Set the option of pre-heat lid at 100 °C
  - 30 °C for 10 min
  - hold at 4 °C

● Protocols

➢ Addition of Index Adapter

- Collect the liquid in the Index Adapter at the bottom by benchtop centrifuge, then mix well by pipetting using 10 μL of pipet with 5 μL setting. Be careful not to cross contaminate between wells.
- Take the LIG2 from storage at -20 °C and place it on a cooler rack (return to -20 °C after use).
- Prepare the RSB and LIG2 cocktail (LIG2 cocktail) as follows. Place the LIG2 cocktail on ice. (RSB 2.5 μL + LIG2 2.5 μL) × (No. of samples + 1)
- Add 5 μL of LIG2 cocktail to each sample. Conduct the experiment on the aluminum PCR tube rack placed on ice. Close the attached cap of each sample's well soon after the addition of LIG2 cocktail to ensure that the LIG2 cocktail is added once to each sample.
- Add 2.5 μL of Index Adapter to each sample (total liquid volume is 37.5 μL). Mix each sample by pipetting (be careful not to raise the air bubble). Spin down using the benchtop centrifuge. Conduct the experiment on the aluminum PCR tube rack placed on ice. Close the attached cap of each sample's well soon after the addition of the adapter to ensure that the adapter is added once to each sample.
- Set the sample tubes on the thermal cycler and run LIG program above (set the liquid amount to 37.5 μL).
- After the program finishes, spin down the solutions using benchtop centrifuge.
- Add 5 μL of STL to each sample (total liquid volume is 42.5 μL).

➢ Clean up Ligated Fragments

- Mix the SPB well by turning the tube upside down and vortexing (ensure that it is well-dispersed).
- Conduct 1st clean up as follows.
  - Add 42.5 μL of SPB to each sample. Mix well by pipetting. Incubate at RT for 5 min.

- Place the sample tubes on the magnetic stand. Incubate for 5 min at RT until the liquid is clear. Remove the supernatant.
- Add 200 μL of 80% EtOH to each sample. Incubate for 30 s at RT. Remove the supernatant.
- Repeat this washing process once again on the magnetic stand.
- Remove the supernatant from the bottom of the tube using 10 μL of pipet. Incubate for 5 min at RT on the magnetic stand (sufficiently dry).
- Add 52.5 μL of RSB to each sample. Take each sample tube from a magnetic stand and mix well by pipetting. Incubate for 2 min at RT.
- Place the sample tubes on the magnetic stand. Incubate for 5 min at RT until the liquid is clear.
- Transfer the 50 μL of supernatant of each sample to the new PCR tube.

- Conduct 2nd clean up as follows.
  - Add 50 μL of SPB to each sample. Mix well by pipetting. Incubate at RT for 5 min.
  - Place the sample tubes on the magnetic stand. Incubate for 5 min at RT until the liquid is clear. Remove the supernatant.
  - Add 200 μL of 80% EtOH to each sample. Incubate for 30 s at RT. Remove the supernatant.
  - Repeat this washing process once again on the magnetic stand.
  - Remove the supernatant from the bottom of the tube using 10 μL of pipet. Incubate for 5 min at RT on the magnetic stand (sufficiently dry).
  - Add 29 μL (28 μL if BioAnalyzer is used for electrophoresis below) of RSB to each sample. Take each sample tube from a magnetic stand and mix well by pipetting. Incubate for 2 min at RT.
  - Place the sample tubes to the magnetic stand. Incubate for 5 min at RT until the liquid is clear.
  - Transfer the 27 μL (26 μL if BioAnalyzer is used for electrophoresis below) of supernatant of each sample to the new 1.5 mL tube (label sample name to each tube).

➢ Electrophoresis (No. 3)

- Conduct electrophoresis using TapeStation (optionally BioAnalyzer) as follows and check the concentration and fragment size of each ligation product. If needed, dilute the supernatant to measure the concentration within the range of quantification to accurately quantify the DNA concentration. If any samples indicate anomalous sizes or concentrations, conduct the electrophoresis again or check if the experimental procedures were conducted appropriately.
- Take the 2 μL portion from the supernatant of each sample and mix well with 2 μL of HSD5000 sample buffer (be careful not to include bubbles and not to use HSD1000).
- Subject each mixture to electrophoresis using TapeStation HSD5000 ScreenTape.

The remaining amount of supernatant of each sample is 25 μL.

To stop the experiment here temporarily (up to 2 days), store samples at -20 °C.

Caution: During the following process, dispense precise liquid amount because the DNA concentration significantly affects conversion efficiency of dsDNA consensus sequence. Especially, during the sample dilution and PCR, be careful not to mix up the samples, the count of serial dilution, and the No. of PCR cycles.

➢ Quantification of DNA concentration

- Quantify the DNA concentration of each sample using TapeStation Analysis Software. The typical fragment size is 250 to 1500 bp.
- Quantify by selecting the overall hump observed in electropherogram of each sample.
- Confirm the shape of the electropherogram, distribution of fragment size, average fragment size, etc. are equivalent between samples.

➢ Dilution of the DNA sample

- Dilute each sample to 400 pM using RSB (400 pM diluent). If the original concentration is lower than 400 pM, dilute to 200 pM and reduce the number of serial dilutions by one.
- Dilute each 400 pM diluents to 3.13 pM (78 attomole/25 μL) by serial dilution as follows. This concentration is important to maximize the conversion rate of consensus sequence per unit initial sequencing amount.
  - Prepare a new eight-strip tube per sample.
  - Add 12.5 μL of RSB each to seven wells of a new eight-strip tube.
  - Add 12.5 μL of 400 pM diluents to the 1st well of the eight-strip tube. Mix well by pipetting (400 pM diluent -> 200 pM diluent).
  - Add 12.5 μL of 200 pM diluents to the next well in the eight-strip tube. Mix well by pipetting (200 pM diluent -> 100 pM diluent).
  - Repeat this dilution process until 3.13 pM concentration is achieved (i.e., 400 -> 200 -> 100 -> 50 -> 25 -> 12.5 -> 6.25-> 3.13).
- Subject 25 μL of each 3.13 pM diluent to PCR enrichment.

To determine the optimal DNA amount subjected to PCR enrichment, prepare several dilutions of ligation products (e.g., 0.8 to 25 pM) and use 25 μL of each diluent for PCR enrichment.

7. Enrichment of DNA fragments by PCR

● Reagents and Setup

- PCR Primer Cocktail (PPC) in TruSeq library prep kit: Thaw at RT. Mix well by turning the tube

upside down several times (no vortex). Then, spin down with a benchtop centrifuge. Store at -20 °C after use.

- Enhanced PCR Mix (EPM): Thaw on ice. Mix well by turning the tube upside down several times (no vortex). Then, spin down using a benchtop centrifuge. Store at -20 °C after use.
- SPB: warm to RT.
- RSB: warm to RT.
- 80% EtOH: freshly prepare just before use (99.5% EtOH:DW = 8:2).

- Setup thermal cycler program for PCR below (PCR nano program).
  - Set the option of pre-heat lid at 100 °C
  - 95 °C for 3 min
  - 15 cycles
    - 98 °C for 20 s
    - 60 °C for 15 s
    - 72 °C for 30 s
  - 72 °C for 5 min
  - hold at 4 °C

- Protocols

➢ Amplify DNA fragments

- Prepare PPC and EPM cocktail as follows. Place the cocktail on ice.
  (PPC 5 µL + EPM 20 µL) × (No. of samples + 1)
- Place the 3.13 pM diluent samples on the aluminum rack on ice. Add 25 µL of PPC and EPM cocktail to each diluent sample. Mix well by pipetting (total liquid volume is 50 µL).
- Spin down using a benchtop centrifuge. Remove air bubble if observed.
- Set the tubes of the diluent samples to the thermal cycler and run PCR nano program above (set the liquid amount to 50 µL).
- After the program finishes, spin down the tube using a benchtop centrifuge.

➢ Clean up Amplified DNA (1st cycle)

- Mix the SPB well by turning the tube upside down and by vortex (well-dispersed).
- Add 50 µL of SPB to each sample. Mix well by pipetting. Incubate for 5 min at RT.
- Place the sample on the magnetic stand. Incubate for 5 min at RT until the liquid is clear. Remove the supernatant.
- Add 200 µL of 80% EtOH to each sample. Incubate for 30 s at RT. Remove the supernatant.
- Repeat this washing process once again on the magnetic stand.

- Remove the supernatant from the bottom of the tube using 10 μL of pipet. Incubate for 5 min at RT on the magnetic stand (sufficiently dry).
- Add 32 μL of RSB to each sample. Take each sample tube from the magnetic stand and mix well by pipetting. Incubate for 2 min at RT.
- Place the sample tubes on the magnetic stand. Incubate for 5 min at RT until the liquid is clear.
- Transfer 30 μL of the supernatant of each sample to the new PCR tube.

➢ Clean up Amplified DNA 2nd cycle

- Mix the SPB well by turning the tube upside down and by vortex (well-dispersed).
- Add 30 μL of SPB to each sample. Mix well by pipetting. Incubate for 5 min at RT.
- Place the samples on the magnetic stand. Incubate for 5 min at RT until the liquid is clear. Remove the supernatant.
- Add 200 μL of 80% EtOH to each sample. Incubate for 30 s at RT. Remove the supernatant.
- Repeat this washing process once again on the magnetic stand.
- Remove the supernatant from the bottom of the tube using 10 μL of pipet. Incubate for 5 min at RT on the magnetic stand (sufficiently dry).
- Add 33 μL of RSB to each sample. Take each sample tube from the magnetic stand and mix well by pipetting. Incubate for 2 min at RT.
- Place the sample tubes on the magnetic stand. Incubate for 5 min at RT until the liquid is clear.
- Transfer 31 μL of the supernatant of each sample to the new 1.5 mL tube (label sample name on each tube).

➢ Electrophoresis (No. 4)

- Conduct electrophoresis using TapeStation (optionally BioAnalyzer) as follows and check the concentration and fragment size of each library's DNA. If needed, dilute the supernatant to measure the concentration within the range of quantification to accurately quantify the DNA concentration. If any samples indicate anomalous sizes or concentrations, conduct electrophoresis again or check if the experimental procedures were conducted appropriately.
- Quantify by selecting the overall hump observed in electropherogram of each sample.
- Take the 1 μL portion from the supernatant of each sample and mix well with 9 μL of RSB (1/10 diluent).
- Take the 2 μL portion from each 1/10 diluent and mix well with 2 μL of HSD1000 sample buffer (be careful not to include air bubbles).
- Subject each mixture to electrophoresis using TapeStation HSD1000 ScreenTape.

The remaining amount of supernatant of each sample is 30 μL.

Store samples at -20 °C.

Caution: If adapter dimers are detected, repeat the cleanup process above (the amount of SPB is 30 μL). Dimers should not be detected as they may decrease the conversion efficiency of the consensus sequence.

8. Pooling of the library DNA

- Precautions
- Check the electropherogram of each library's DNA and confirm that the shape, fragment distribution, average fragment size, etc. are equivalent between samples.
- Prepare the mixture of the library DNAs (lib-mix) as follows. The concentration of the lib-mix should be over 10 nM, and the volume should be over 20 μL (over 30 μL if sequence over 600 Gbp).

- Protocols
- Based on the concentration measured in electrophoresis No. 4, adjust each library DNA sample concentration to 10 nM using RSB (10 nM library DNA sample).
- Add an equal amount of each 10 nM library DNA sample to a single 1.5 mL tube and mix well by pipetting.
  e.g., during the pooling of six library DNAs, add 4 μL of each 10 nM library DNA sample to a single tube to prepare 24 μL of library pool.
- Label the tube with the name of the library pool, and store at -20 °C.

Appendix:

Reagents and protocol for electrophoresis using Agilent 2100 BioAnalyzer (Agilent technologies, CA, USA)

- Reagents for 2100 BioAnalyzer
  - High-Sensitivity DNA (HSD) kit: warm to RT before use.

- Protocol
  - Take the 1 μL portion from the supernatant of each sample and subject it to electrophoresis using BioAnalyzer HSD.
  - In electrophoresis No.3, quantify the DNA concentration of each sample using Region Table. The typical fragment size is 400 to 2000 bp (for detail, look at 2100 expert software user’s guide).
  - Quantify by selecting the overall hump observed in the electropherogram of each sample.
  - In electrophoresis No.4, take the 1 μL portion from the supernatant of each sample and mix well

with 19 µL of RSB (1/20 diluent).

- Take the 1 µL portion from each 1/20 diluent and subject it to electrophoresis using BioAnalyzer HSD.

Example of electropherogram (electrophoresis No. 1 to No. 4)

Electrophoresis No. 1

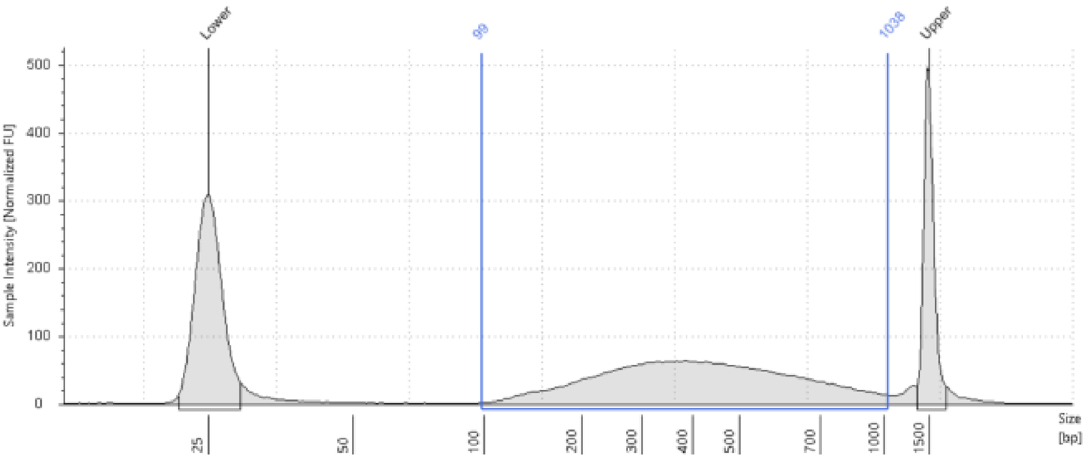

Region Table

From [bp]	To [bp]	Average Size [bp]	Conc. [pg/µl]	Region Molarity [pmol/l]	% of Total	Region Comment	Color
99	1038	434	652	2930	89.11		

Electrophoresis No. 2

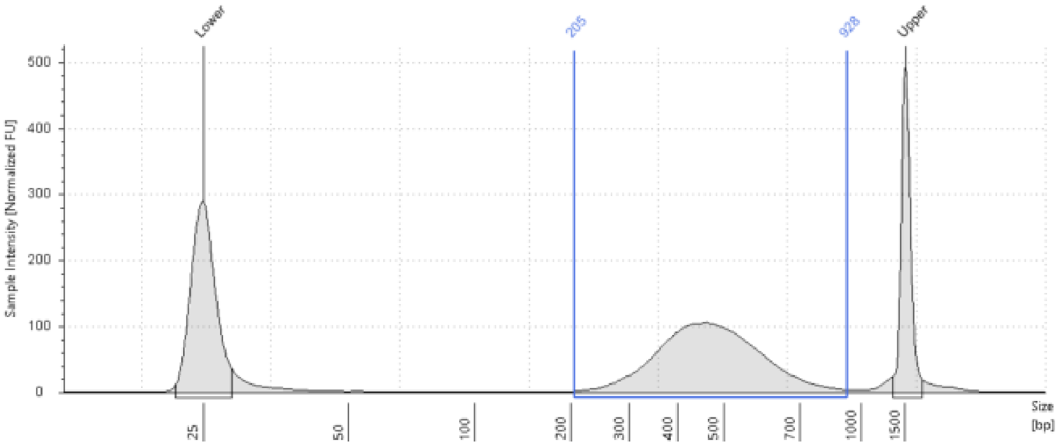

Region Table

From [bp]	To [bp]	Average Size [bp]	Conc. [pg/µl]	Region Molarity [pmol/l]	% of Total	Region Comment	Color
205	928	478	582	2000	88.73		

Electrophoresis No. 3

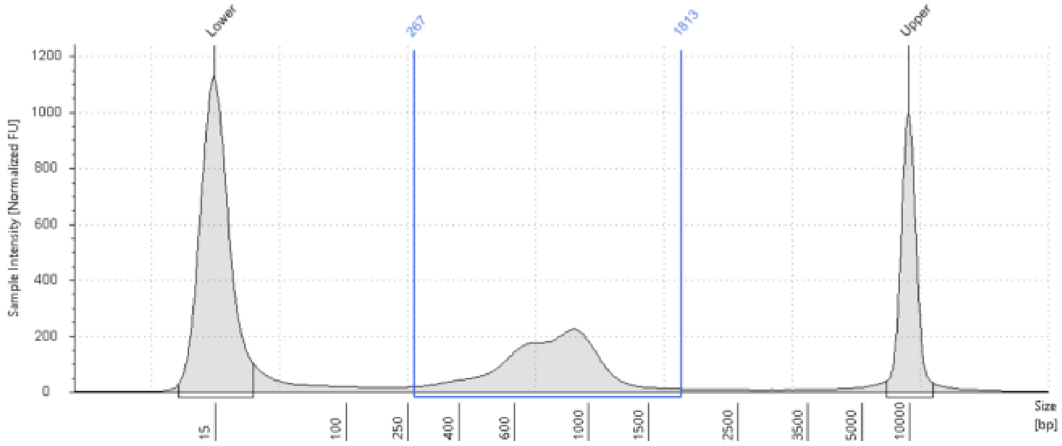

Region Table

From [bp]	To [bp]	Average Size [bp]	Conc. [pg/µl]	Region Molarity [pmol/l]	% of Total	Region Comment	Color
267	1813	798	268	585	76.21		

Electrophoresis No. 4

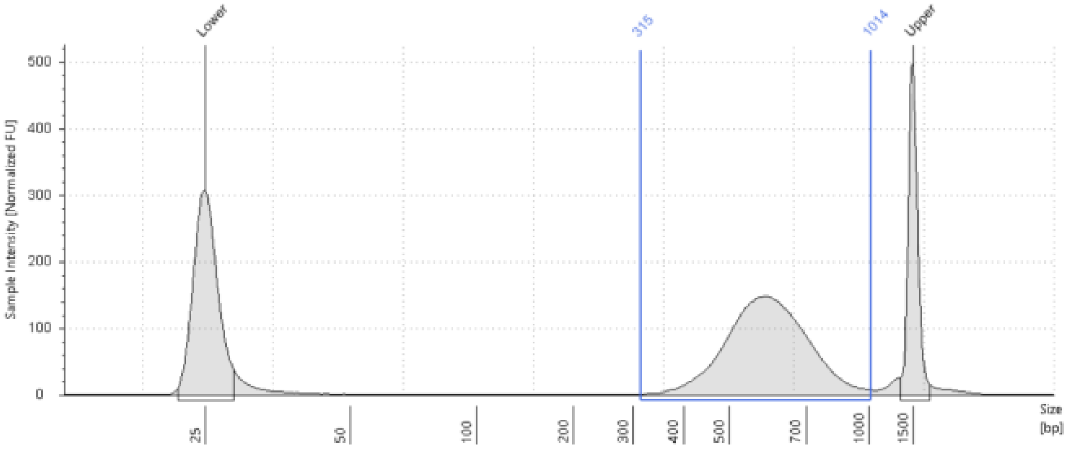

Region Table

From [bp]	To [bp]	Average Size [bp]	Conc. [pg/µl]	Region Molarity [pmol/l]	% of Total	Region Comment	Color
315	1014	607	623	1650	91.18		
